# Copper–Zinc‐Doped Bilayer Bioactive Glasses Loaded Hydrogel with Spatiotemporal Immunomodulation Supports MRSA‐Infected Wound Healing

**DOI:** 10.1002/advs.202302674

**Published:** 2023-11-30

**Authors:** Shicheng Huo, Shu Liu, Qianqian Liu, En Xie, Licai Miao, Xiangyu Meng, Zihao Xu, Chun Zhou, Xuesong Liu, Guohua Xu

**Affiliations:** ^1^ Department of Orthopedic Surgery Spine Center Changzheng Hospital Navy Medical University Shanghai 200003 China; ^2^ Department of Spine Surgery Changhai Hospital Navy Military Medical University 168 Changhai Road Shanghai 200433 China; ^3^ Department of Medical Record Statistics Sichuan Provincial People's Hospital University of Electronic Science and Technology of China Chengdu China; ^4^ Key Laboratory for Ultrafine Materials of Ministry of Education East China University of Science and Technology Shanghai 200237 China; ^5^ Department of Orthopedics Trauma Shanghai Changhai Hospital Naval Medical University Shanghai 200433 China; ^6^ Orthpaedic Trauma Department of Orthopedics Renji Hospital School of Medicine Shanghai Jiao Tong University Shanghai China; ^7^ Department of Ultrasound Renji Hospital School of Medicine Shanghai Jiao Tong University Shanghai China

**Keywords:** bioactive glass, copper, immunomodulation, infection‐related wounds, zinc

## Abstract

Developing biomaterials with antimicrobial and wound‐healing activities for the treatment of wound infections remains challenging. Macrophages play non‐negligible roles in healing infection‐related wounds. In this study, a new sequential immunomodulatory approach is proposed to promote effective and rapid wound healing using a novel hybrid hydrogel dressing based on the immune characteristics of bacteria‐associated wounds. The hydrogel dressing substrate is derived from a porcine dermal extracellular matrix (PADM) and loaded with a new class of bioactive glass nanoparticles (BGns) doped with copper (Cu) and zinc (Zn) ions (Cu–Zn BGns). This hybrid hydrogel demonstrates a controlled release of Cu^2+^ and Zn^2+^ and sequentially regulates the phenotypic transition of macrophages from M1 to M2 by alternately activating nucleotide‐binding oligomerization domain (NOD) and inhibiting mitogen‐activated protein kinases (MAPK) signaling pathways. Additionally, its dual‐temporal bidirectional immunomodulatory function facilitates enhanced antibacterial activity and wound healing. Hence, this novel hydrogel is capable of safely and efficiently accelerating wound healing during infections. As such, the design strategy provides a new direction for exploring novel immunomodulatory biomaterials to address current clinical challenges related to the treatment of wound infections.

## Graphical abstract

The advancement of biomaterials possessing both antimicrobial and wound‐healing properties for the treatment of wound infections continues to pose a formidable challenge. The present study introduces a novel sequential immunomodulation strategy predicated on the immune characteristics of bacterial‐associated wounds. This approach employs an innovative hybrid hydrogel dressing designed to expedite the healing process of infected wounds effectively and rapidly.

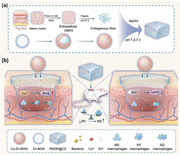

## 1 Introduction

The skin is the largest organ in the body and serves as the first line of defense against the external environment.^[^ ^1^ ^]^ Meanwhile, microorganisms that breach this barrier via infection of wounds induce severe inflammation, negatively impacting healing and potentially causing life‐threatening conditions.^[^ ^2^ ^]^ It is therefore essential to develop novel multifunctional bioactive materials capable of eliminating infections and promoting wound healing and tissue regeneration. Accordingly, several wound‐dressing materials, including nanofibrous membranes and hydrogels, have been developed.^[^ ^3^ ^]^

Hydrogels are highly permeable, biocompatible materials with hydrating properties. As such, they are promising candidates for healing wounds caused by infections.^[^ ^4^ ^]^ However, given that conventional hydrogel dressings lack antimicrobial activity, they are often loaded with antibiotics, which can lead to bacterial resistance and hepatic/nephrotoxicity, ultimately impairing wound healing.^[^ ^5^ ^]^ Hence, an urgent need exists for the development of novel antibiotic‐free hydrogel wound dressings.

Macrophages mediate immune responses, antibacterial activity, and wound healing.^[^ ^6^ ^]^ Moreover, they exhibit phenotypic plasticity and can be categorized as M1 (proinflammatory) or M2 (anti‐inflammatory) based on their responses to diverse microenvironments.^[^ ^7^ ^]^ Microbial infections accompanied by biofilm formation favor the polarization of macrophages toward the M2 phenotype. However, this creates an immunosuppressive microenvironment that fails to eliminate bacteria and can inhibit wound healing.^[^ ^8^ ^]^ Therefore, reversing the immunosuppressive microenvironment of the infected skin is necessary to ensure bacterial clearance and wound healing. During the early stages of infection, M1 macrophage polarization inhibits bacterial growth and eliminates infections,^[^ ^8^, ^9^ ^]^ which also effectively reduces biofilm formation.^[^ ^10^ ^]^ Therefore, targeting proinflammatory macrophages could reverse immunosuppression and promote antimicrobial activity. Indeed, an early inflammatory response is essential for preventing infection, removing tissue debris, and wound healing.^[^ ^11^ ^]^ Meanwhile, prolonged M1 macrophage dominance results in excessive or chronic inflammation, which damages tissues, accelerates scar formation, and impedes wound healing.^[^ ^12^ ^]^ Accordingly, during tissue repair, following an early transitional inflammatory phase, macrophages switch to the M2‐type. In addition to suppressing M1 macrophages, anti‐inflammatory factors such as interleukin (IL)−4 and IL‐10 secreted by M2 macrophages promote tissue repair and maturation, as well as the stabilization of vessels, thereby accelerating the process of wound healing.^[^ ^13^ ^]^ Hence, early onset inflammation promotes the clearance of infection and tissue repair, while wound healing requires a timely shift from an activated (M1) to an alternative (M2) macrophage response. Therefore, sequential immunomodulatory strategies are required for bacterial clearance, wound healing, and tissue regeneration. As such, during the early stages of infection‐induced skin injury, biomaterials should induce M1 polarization to reduce bacteria proliferation and facilitate tissue repair. However, in the later stages, M2 macrophage polarization must be stimulated to promote wound healing. Hence, it is crucial to develop innovative biomaterials that align with this physiological process in order to enhance the efficacy of infection‐related wound healing.

Trace elements like copper (Cu) and zinc (Zn) are critically involved in cell metabolism, tissue regeneration, and immunity.^[^ ^8^, ^14^ ^]^ In fact, Cu^2+^ possesses strong bactericidal and antimicrobial activities against diverse bacteria, including antibiotic‐resistance bacteria.^[^ ^15^ ^]^ In addition, Cu^2+^ secretes pro‐inflammatory factors, thereby promoting the formation of a pro‐inflammatory microenvironment.^[^ ^9^ ^]^ In contrast, Zn^2+^ has good angiogenic and tissue‐repair properties. Moreover, Zn^2+^ interacts with immune cells to facilitate the formation of an anti‐inflammatory microenvironment.^[^ ^16^ ^]^ Considering that Cu^2+^ and Zn^2+^ regulate the immune responses in different ways, their sequential controlled release of both these ions at different stages of injury, that is, the release of Cu^2+^ during the early stage and Zn^2+^ during the later stage, could exert dual‐temporal bidirectional immunoregulatory functions.

**Scheme 1:**
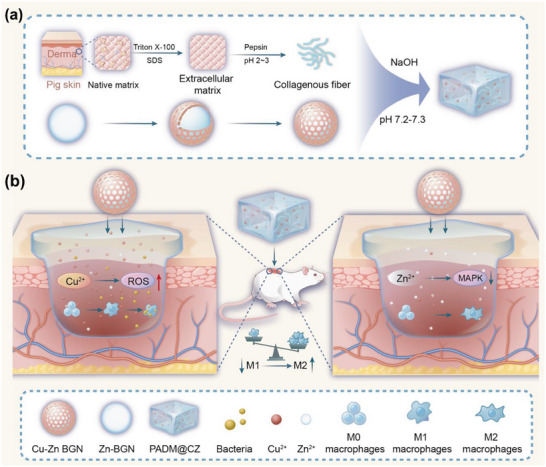
a) Schematic of PADM@CZ hydrogel synthesis and b) the dual‐temporal bidirectional immunomodulatory effects of PADM@CZ.

In this study, we designed and synthesized an innovative hydrogel comprising a Zn‐containing bioactive glass nanoparticle (Zn‐BGn) core covered with a porous layer of Cu‐containing bioactive glass, designated Cu–Zn BGn. During the early stages, the outer Cu layer of the Cu–Zn BGn releases Cu^2+^ to induce a proinflammatory response, thereby inhibiting infection. At the later stages, the Zn‐containing core releases Zn^2+^ to form an anti‐inflammatory microenvironment and promote tissue repair. Moreover, Cu–Zn BGns are uniformly loaded into a porcine dermal extracellular matrix‐derived hydrogel (PADM@CZ, **Scheme** **1**). The results revealed that the PADM@CZ hydrogel significantly reduced the toxic effects of nanoparticles and accelerated wound healing. Because molecules on the extracellular matrix (ECMs) mimic the cellular microenvironment, integrating mechanical and chemical cues could aid in the adhesion, proliferation, and differentiation of cells, thus facilitating homologous wound healing.^[^ ^17^ ^]^ Hence, the PADM@CZ hydrogel has the potential to exhibit superior antimicrobial and immunomodulatory properties and improved bacterial clearance, thus, promoting wound healing by reshaping immune homeostasis.

## 2 Results and Discussion

### 2.1 Characterization of Hydrogel

TEM images revealed the apparent spherical core–shell structure of the Cu–Zn BGns, with Cu ions dominating the outer layer as identified by energy dispersive spectroscopy (EDS), and Zn ions primarily located in the inner layer (Figure S1a,b, Supporting Information). Dynamic light scattering (DLS) analysis confirmed that the Cu–Zn BGns had a narrow size distribution with an average hydrodynamic diameter of 229.8 nm (Figure S1c, Supporting Information).

**Figure 1:**
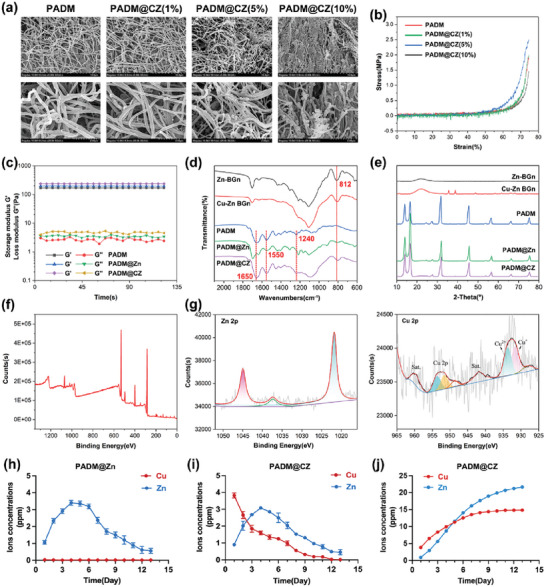
Characterization of Cu–Zn BGns and PADM@CZ hydrogel. a) SEM images of various PADM@CZ hydrogels (1%, 5%, and 10%). b) Typical compressive stress–strain curves of various PADM@CZ hydrogels (1%, 5%, and 10%). c) Storage modulus (*G*′) and loss modulus (*G*′′) of PADM, PADM@Zn, and PADM@CZ hydrogels. d) FTIR spectra, e) XRD patterns of Zn‐BGns, Cu‐Zn‐BGns, and various hydrogels. XPS spectra of the fully scanned region f) and the g) Zn 2p region and Cu 2p region of PADM@CZ hydrogels. h,i) Concentration of released Cu^2+^ and Zn^2+^ of the h) PADM@Zn and i) PADM@CZ. j) Cumulative release of Cu^2+^ and Zn^2+^ in PADM@CZ.

Hematoxylin and eosin (HE) and 4′6‐diamino‐2‐phenylindole (DAPI) staining confirmed the complete removal of porcine skin cells with an intact extracellular structure (Figure S2a,b, Supporting Information), consistent with a previous study.^[^ ^17^ ^]^ Representative SEM images of PADM and PADM@CZ (1%, 5%, and 10%) are presented in **Figure** **1**a. Similar to the ECM and tendon hydrogels, the PADM and PADM@CZ hydrogels had a reticular and closely packed fiber architecture, thereby indicating that extracellular collagen digested by pepsin could reassemble into collagen‐like fibers.^[^ ^18^ ^]^ In addition, the SEM results confirmed the incorporation of Cu–Zn BGns into the PADM@CZ hydrogel. However, at higher particle concentrations, particularly at 10%, the original structure of the PADM became obscured, which could potentially impact its functionality. Consequently, we tested the mechanical properties of hydrogels at varying particle concentrations (Figure 1b). Hydrogels incorporating 1% and 5% Cu–Zn BGns, particularly the latter, exhibited superior mechanical strength compared to pure PADM hydrogels. However, the inclusion of 10% particles reduced the mechanical strength of the PADM hydrogels. Consequently, PADM@CZ (5%) was selected for subsequent analysis. Additionally, throughout the rheometer test, the storage modulus (*G*′) of the hydrogels remained constant and consistently higher than their loss modulus (*G*′′), indicating stable gelation and favorable mechanical properties (Figure 1c).

Fourier‐transform infrared analysis (FTIR) was employed to characterize the PADM and PADM@CZ hydrogels (Figure 1d). FITR spectra of the PADM hydrogels showed peaks corresponding to collagen fibers within the PADM, including amide I at 1650 cm^−1^, II at 1550 cm^−1^, and III at 1240 cm^−1^.^[^ ^19^ ^]^ The FTIR spectrum of the PADM@CZ hydrogel showed no noticeable differences or shifts in the bands, specifically the amide I band corresponding to the triple helical morphology of the collagen. The characteristic peaks of BGns were at 812 cm^−1^ due to the symmetric stretching vibrations band of Si─O─Si, and at 1299–900 cm^−1^ due to the asymmetric vibration bands of Si─O─Si (bridging bonds) and the Si─O─ (non‐bridging bonds).^[^ ^20^ ^]^ Therefore, the FTIR spectra validated the successful fabrication of PADM@CZ hydrogels. XRD analysis also provides information on the axial arrangement of collagen fibrils and the three‐stranded helical conformation of the molecules.^[^ ^21^ ^]^ Meanwhile, the PADM is primarily composed of type I collagen. The XRD patterns (Figure 1e) of the PADM before and after incorporating Cu–Zn BGns showed similar curves. In addition, no significant changes were observed in the shape or positions of the three diffraction peaks specific to PADM. The XRD results revealed that the incorporation of Cu–Zn BGns into the PADM hydrogel did not deteriorate the natural conformation, retaining the biological properties of PADM. Moreover, X‐ray photoelectron spectroscopy (XPS) suggested the presence of Cu and Zn elements in the C, N, and O matrices of PADM, indicating the incorporation of Cu–Zn BGns (Figure 1f). The relatively low XPS spectra of Cu 2p and Zn 2p showed that the Cu–Zn BGns were embedded into the inner framework of the PADM@CZ hydrogel (Figure 1g), which could not be fully confirmed by XPS surface element analysis.

The water retention properties of the PADM and PADM@CZ hydrogels are presented in Figure S2c (Supporting Information). Both hydrogels lost all their water content after approximately 30 h at the restrictive temperature (37°C), with no significant differences between them. Thus, incorporating the Cu–Zn BGns did not alter the water‐retention properties of the PADM hydrogel. Hence the PADM hydrogel should be capable of retaining moisture in the wound and promoting wound healing for a longer duration.^[^ ^22^ ^]^ Furthermore, the hydrogels might have the capacity to absorb some of the leaked fluids from the wound defects, thereby promoting wound healing.^[^ ^23^ ^]^

Moreover, an increased rate of PADM and PADM@CZ hydrogel degradation was observed with increasing temperature (Figure S2d, Supporting Information). Both hydrogels completely degraded when incubated at 42 °C for 2 d. However, at the same time points, the blank and PADM@CZ hydrogels at 25 °C and 37 °C retained 0.46 and 0.54 g, respectively. Next, the hydrogels were digested with trypsin to analyze the rate of hydrogel degradation. Figure S2e (Supporting Information) shows the minimum difference in the rates of PADM and the PADM@CZ hydrogel degradation in trypsin and phosphate‐buffered saline (PBS). The similarity in the degradation rates can be attributed to the fact that Cu–Zn BGns and PADM hydrogels primarily interact via adsorption and the enzymes failed to degrade nanoparticles.^[^ ^24^ ^]^ However, a significant increase in the rate of hydrogel degradation was observed with trypsin compared with PBS alone. This can be attributed to the decomposition of decellularized porcine skin into collagen fibers during hydrogel preparation and the presence of proteins degraded by trypsin in the PADM hydrogel.^[^ ^25^ ^]^ These results indicate that the proteins in hydrogels can be degraded by locally secreted proteases, thereby promoting the secretion of the active ingredients.

Indeed, Cu^2+^ and Zn^2+^ were sequentially released from the PADM@CZ hydrogels (Figure 1h,i). The Cu^2+^ concentration was higher during the early stage, specifically on Day 1, compared to Zn^2+^. However, after Day 2, the Zn^2+^ concentration was higher than that of Cu^2+^. Notably, it was not until day 5 that the cumulative concentration of Cu^2+^ slightly decreased below that of Zn^2+^ (Figure 1j). Hence, upon exposure to the solution, the outer porous layer of Cu degraded, followed by slow degradation of the dense Zn layer.

### 2.2 Antibacterial Activity and Mechanisms of PADM@CZ Hydrogel

**Figure 2:**
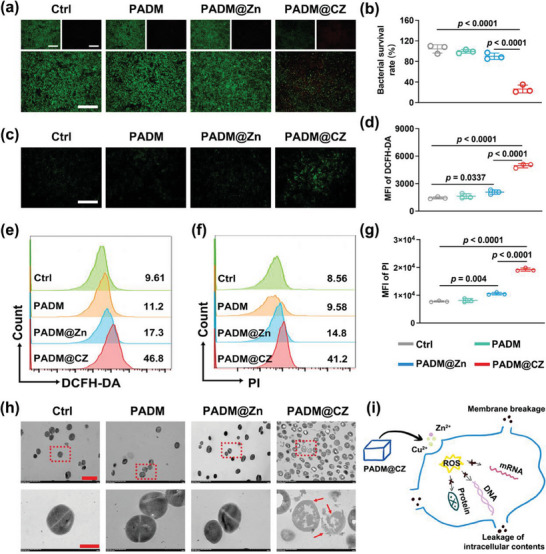
In vitro antibacterial property of PADM@CZ. a) MRSA viability following different interventions using live/dead staining. The green fluorescence is indicative of live bacteria while the red fluorescence represents dead bacteria. Scale bar: 100 µm. b) Survival rates of MRSA following incubation with different treatments. Scale bar: 100 µm. c–e) Assessment of ROS generation was conducted using DCFH‐DA fluorescent probes. f,g) Detection of membrane damage in MRSA resulting from different treatments via monitoring PI influx. h) MRSA morphology was examined through representative TEM images after employing different treatments. The red arrow signifies the compromised bacterial cell membrane. Scale bar: 20 µm (upper panel); 500 nm (lower panel). The depicted schematic offers insights into the antibacterial properties of PADM@CZ against MRSA. b,d,g) Data are presented as mean ± SD (*n =* 3 per group), with “*n*” denoting biologically independent experiments.

Methicillin‐resistant *Staphylococcus aureus* (MRSA) was used to test the antibacterial properties of the PADM@CZ hydrogel in vitro. First, the growth curves of the bacteria were plotted under different conditions based on the absorbance of the bacterial suspension at the onset. The results revealed a significant reduction in the amplification rates of bacteria treated with the PADM@CZ hydrogel. However, treatment with the PADM@Zn hydrogel yielded limited inhibitory effects on bacterial growth (Figure S3, Supporting Information). Next, cells from different treatment groups were stained with live/dead dyes and observed under a fluorescence microscopy (**Figure** **2**a). Significant cell death and red staining of MRSA were observed in the PADM@CZ group. Furthermore, relatively few dead bacterial cells were observed in the control and PADM@Zn groups, thereby indicating that the PADM@Zn hydrogel exerted a weak bactericidal effect. Next, we used the spread‐plate method (SPM) to quantify the colonies. The survival rate of bacteria in the PADM@CZ group decreased from 100.15% ± 6.45% to 23.85% ± 6.39%, whereas no significant reduction in the survival rate was observed in the other groups (Figure 2b; Figure S4a, Supporting Information). These results revealed that the PADM@CZ hydrogel exhibited a strong bactericidal activity, which was primarily attributed to the presence of Cu^2+^ rather than Zn^2+^.

A previous study has shown that Cu^2+^ uses several mechanisms to kill bacteria, including the production of reactive oxygen specifies (ROS) to disrupt cell membranes, inhibit metabolic processes, and induce DNA fragmentation to kill the bacteria.^[^ ^26^ ^]^ Therefore, we first determined the involvement of ROS in the antibacterial activity using dichloride‐fluorescein diacetate (DCFH‐DA) to identify the underlying bactericidal mechanism of the PADM@CZ hydrogels. Flow cytometry results showed that the PADM@CZ hydrogel increased ROS levels in the bacteria (Figure 2e). The intensity of DCFH‐DA fluorescence in the PADM@CZ group was 2.1‐, 3,3‐, and 3.6‐fold higher than that in the other groups, respectively. These results were validated by fluorescence microscopy (Figure 2c,d). Next, we used propidium iodide (PI, a nucleic acid‐binding dye) to determine whether the PADM@CZ hydrogel could disrupt cell membranes. The results revealed that PADM@CZ hydrogel significantly increased the permeability of the bacterial cell membranes (Figure 2f).

In the PADM@CZ group, the PI intensity increased by 32.64%, 31.62%, and 27.6%, respectively, compared with the bacteria in the control, PADM, and PADM@Zn groups, respectively. Hence, the PADM@CZ hydrogel treatment significantly disrupted the cell membranes of the bacteria. Finally, we quantified the fluorescence intensity of PI using a fluorescence microplate reader; the results were consistent with those of flow cytometry (Figure 2g). Additionally, MRSA was treated with different hydrogels, and the absorbances of nucleic acids and proteins were measured at 260 nm (A260) and 280 nm (A280) to determine cell leakage. A detectable increase in the absorbance of nucleic acids and proteins at 260 nm and 280 nm was observed in the PADM@CZ group compared to the control, PADM, and PADM@Zn groups (Figure S4b, Supporting Information). Furthermore, TEM images showed shrunken, blurred borders and crumpled appearance of bacterial membranes in the PADM@CZ group. Smooth, intact, and well‐defined cell membranes were observed in the control, PADM, and PADM@Zn groups (Figure 2h).

These results demonstrated that the PADM@CZ hydrogels exerted a good anti‐bacterial effect in vitro. That is, the metal ions are degraded and continuously released by the PADM@CZ hydrogels in an acidic microenvironment during bacterial infection. During the early stages of infection, Cu^2+^ is predominantly released, along with trace amounts of Zn^2+^. These metal ions, specifically Cu^2+,^ act as powerful bactericidal agents and disrupt bacterial cell membranes, resulting in leakage of cellular contents and alterations in biochemical processes. Subsequent induction of ROS production induces DNA damage, leading to cell death (Figure 2i).

### 2.3 Immunomodulatory Role of the PADM@CZ Hydrogel

Macrophages are important effectors of the innate immune system and are involved in immune regulation and elimination of microbes, thereby defending the body against infections.^[^ ^6^, ^27^ ^]^ Studies have shown that polarized macrophages are divided into M1 and M2 types.^[^ ^28^ ^]^ Proinflammatory M1 macrophages aid in forming a proinflammatory microenvironment by producing several proinflammatory cytokines, including tumor necrosis factor‐α (TNF‐α), IL‐6, and specific surface markers or proteins (CCR7 and inducible nitric oxide synthase [iNOS]).^[^ ^29^ ^]^ In contrast, M2 macrophages create an anti‐inflammatory microenvironment by secreting anti‐inflammatory cytokines, such as IL‐4, CD206, and IL‐10.^[^ ^30^ ^]^ Meanwhile, bacterial biofilm secretions, including extracellular polymers, etc., can impede bacterial clearance and prevent the M1 polarization of macrophages, thereby suppressing (or silencing) the immune response.^[^ ^31^ ^]^ Additionally, excess toxins secreted by bacteria are detrimental to the activity and function of macrophages, suppressing local immune responses against infections.^[^ ^32^ ^]^ Thus, reversing the immunosuppressive microenvironment at the infection site by stimulating M1 macrophage polarization may be a promising approach for eliminating infections.

We next explored the immunomodulatory effects of the various hydrogels on macrophages. In the PADM@CZ group, a significant increase in the secretion of IL‐6 and TNF‐α by macrophages was observed on Days 1–7, and a decrease in the secretion of IL‐1β and IL6 was observed on Days 9–13 (Figure 5a,b). Furthermore, an increase in the secretion of IL‐4 and IL‐10 was observed on Days 9–13 in the PADM@CZ group (Figure S5c,d, Supporting Information). These results demonstrated that the PADM@CZ hydrogel could transition from a pro‐ to an anti‐inflammatory immune response over time.

**Figure 3:**
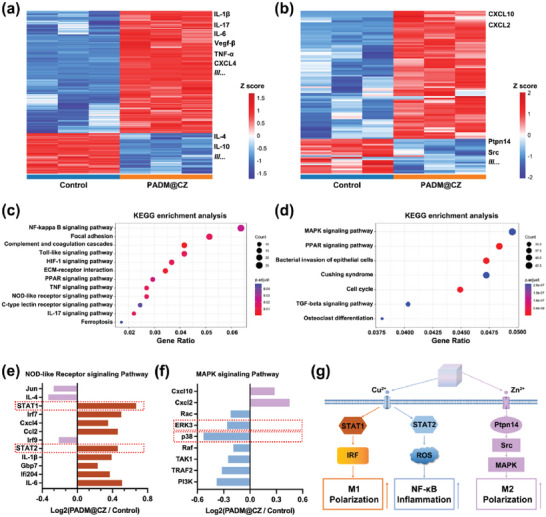
Gene expression analysis of RAW 264.7 macrophages in response to the PADM@CZ. a,b) Microarray heat map visualizing the fold change in expression of cell‐specific genes co‐cultured with PACM@CZ samples on Days a) 5 and b) 11. c) Pathways upregulated in cells co‐cultured with PACM@CZ samples on Day 5 were analyzed using the KEGG pathway method. d) Pathways downregulated in cells co‐cultured with PACM@CZ samples on Day 11 were analyzed using the KEGG pathway method. e) Fold change in the expression of genes related to the NOD‐like receptor signaling pathway in cells co‐cultured with PACM@CZ samples on Day 5. f) Fold change in the expression of genes related to the MAPK signaling pathway in cells cocultured with PACM@CZ samples on Day 11. g) Schematic illustration of the dual‐temporal bidirectional immunomodulatory effects of PADM@CZ.

RNA‐sequencing (RNA‐seq) was performed on RAW 264.7 cells treated with Day 5 and 11 PADM@CZ hydrogel extracts for 24 h. Correlation analysis showed a high level of consistency among samples in each treatment group, suggesting comparable gene expression between the groups (Figure S6a,d, Supporting Information). We then screened for differentially expressed genes (DEG) as per the following criteria: *P* < 0.05 and fold change (FC) ≥ 2. A total of 1028 DEGs were identified in the Day 5 PADM@CZ group, compared to the control group, of which 743 were upregulated, and 285 were downregulated (Figure S6c, Supporting Information). In the Day 11 PADM@CZ group, 199 DEGs were identified, of which 95 were upregulated and 104 were downregulated (Figure S6f, Supporting Information). Finally, heat maps of the DEGs from the Day 5 PADM@CZ and Day 11PADM@CZ groups compared to the corresponding control groups were constructed (**Figure** **3**a,b), revealing an increase in *CCR7* and *iNOS* (M1 macrophage markers) expression and a decrease in the expression of *CD206* and *IL‐10* (M2 macrophage‐related genes) in the Day 5 PADM@CZ group. In contrast, in the Day 11 PADM@CZ group, an increase in *CD206* and *IL‐10* and a decrease in *CCR7* and *iNOS* expression was observed, indicating that macrophages treated with PADM@CZ hydrogels were polarized to an anti‐inflammatory M2 type.

Next, we performed the Kyoto Encyclopedia of Genes and Genomes (KEGG) pathway enrichment analysis. Results showed that pathways regulating macrophage polarization were enriched in cells treated with the PADM@CZ hydrogel. Specifically, M1 pathways, namely, the NOD‐like and NF‐κB signaling pathways, were upregulated significantly after treatment with the Day 5 PADM@CZ sample (Figure 3c). In addition, the TNF signaling pathway was significantly upregulated. Meanwhile, in macrophages of the Day 11 PADM@CZ group, a decrease in the MAPK pathway, which regulates M2 polarization, was observed, indicating polarization toward the M2 phenotype (Figure 3d). Thus, the microarray results revealed that the PADM@CZ hydrogel could induce M1 macrophage polarization at an early stage and induces M2 macrophage‐specific gene expression at later stages.

**Figure 4:**
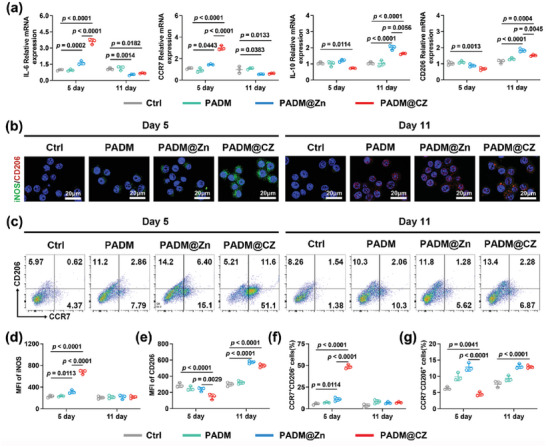
The potential immunomodulatory impact of PADM@CZ hydrogels on macrophages. a) RT‐PCR analysis results of *IL‐6*, *CCR7*, *IL‐10*, and *CD206*. b) Immunofluorescent staining on RAW264.7 cells that were cultured in extracts from Days 5 and 11 samples for iNOS (green), CD206 (red), and DAPI (blue). Scale bar: 20 µm. c) Scatter plots depicting the RAW264.7 cellular surface markers CCR7 (indicative of M1 macrophages) and CD206 (demonstrative of M2 macrophages) were analyzed via flow cytometry. Quantification of the ratio of d) iNOS and e) CD206 positive cells accomplished via immunofluorescent staining. Positive f) CCR7 and g) CD206 cell proportion. a,d,e–g) Data are presented as mean ± SD (*n =* 3 per group), with “*n*” denoting biologically independent experiments.

We performed real‐time polymerase chain reaction (RT‐PCR) to determine the expression of several genes and validate the immunomodulatory effects of the PADM@CZ hydrogel (**Figure** **4**a). RT‐PCR results revealed a significant increase in the expression of *IL‐6*, *CCR‐7*, and *iNOS*, and a decrease in that of *CD206* and *IL‐10*, in macrophages cultured with Day 5 PADM@CZ hydrogel. However, the opposite results were observed for the macrophages co‐cultured with the Day11 PADM@CZ hydrogel. That is, a significant decrease in the expression of M1 macrophage‐related genes and an increase in the expression of M2 macrophage‐related genes were observed. Immunofluorescence and flow cytometry were performed to investigate macrophage phenotypes (Figure 4b–g). A pro‐inflammatory microenvironment was established on Day 5 using the PADM@CZ hydrogel by activating M1 macrophage polarization and stimulating cytokine secretion. On Day 11, M2 macrophage polarization and anti‐inflammatory cytokine secretion were induced in PADM@Zn and PADM@CZ hydrogels. Furthermore, the PADM@CZ hydrogel regulated M1 macrophage polarization to M2 from Day 5 to 11, demonstrating dual‐temporal bidirectional immunomodulatory effects. These results highlighted the potential use of the PADM@CZ hydrogel for preventing infections and promoting tissue regeneration.

The PADM@CZ hydrogel exhibited a dual‐temporal bidirectional immunomodulatory activity in response to varying Cu^2+^ and Zn^2+^ concentrations over time. However, the mechanisms underlying the regulation of regulating macrophage phenotypes by Cu^2+^ required further investigation. Cu^2+^ reportedly promotes M1 macrophage polarization and proinflammatory cytokine secretion.^[^ ^33^ ^]^ Interestingly, several studies have analyzed the response of macrophages to Cu^2+^; however, the conclusions are conflicting. These discrepancies are likely primarily attributed to the Cu^2+^ concentration. For instance, Storkánová et al. showed an increase in the expression of *IL‐10* and *CD206* after treatment with low Cu^2+^ concentrations (<0.64 ppm). O In contrast, a high Cu^2+^ concentration promotes the secretion of proinflammatory factors, such as *TNF‐α* and *CCR7*, suggesting macrophage polarization toward the M1‐type.^[^ ^34^ ^]^ In the current study, macrophages treated with Day 5 PADM@CZ hydrogels (approximately 1.4 ppm Cu^2+^), exhibited an increase in M1‐macrophage‐related markers, which is consistent with the relationship between the presence of Cu^2+^ and its effect on M1 macrophages.

In contrast, Zn^2+^ induces M2 macrophage polarization and the secretion of anti‐inflammatory cytokines.^[^ ^35^ ^]^ However, Zn^2+^ exerts immunomodulatory effects in a concentration‐dependent manner. High Zn^2+^ concentrations (>65.13 ppm) promoted M1 macrophage activation, whereas low Zn^2+^ concentrations promoted the secretion of anti‐inflammatory cytokines and inhibited proinflammatory cytokine expression. A similar phenomenon was observed in the present study. PADM@CZ hydrogels demonstrated slight proinflammatory effects on Day 5 (2.8 ppm Zn^2+^), however, failed to mediate strong proinflammatory effects, like Cu^2+^. Furthermore, on Day 11 (0.83 ppm), as the Zn^2+^ concentration decreased in PADM@CZ hydrogels, a significant increase in the secretion of anti‐inflammatory cytokines was observed.

### 2.4 Macrophage‐Mediated Antibacterial Activity

**Figure 5:**
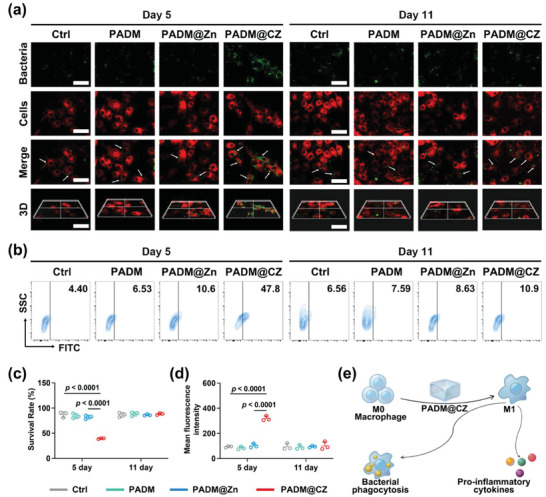
Assessment of the bactericidal impacts of different groups on MRSA through macrophage‐mediated mechanisms. a) Representative fluorescence staining of the bacterial phagocytosis formed by the MRSA (green; the white arrow points to the bacteria inside the cell) phagocytosed by macrophages (red) cultured with various samples (left panel: Day 5 hydrogel extracts; right panel: Day 11 hydrogel extracts). Scale bar: 40 µm. b) Flow cytometry plots depicting the phagocytic clearance of bacteria by macrophages cultured with various samples (left panel: Day 5 hydrogel extracts; right panel: Day 11 hydrogel extracts). c) Quantitative analysis of MRSA colonies that had survived phagocytosis by macrophages. d) Quantification of fluorescence intensity after phagocytosis of MRSA by macrophages cultured with various samples. e) Schematics illustrating the ability of PADM@CZ to sustain the proinflammatory phenotypes of macrophages. c,d) Data presented are expressed as mean ± SD (*n =* 3 per group), with “*n*” denoting biologically independent experiments.

Given that macrophages eliminate infections and necrotic tissues and induce tissue repair via phagocytosis,^[^ ^36^ ^]^ we investigated the effects of the PADM@CZ‐activated macrophages on bacteria. To evaluate the effect of PADM on macrophage phagocytosis, CFDA‐SE‐labeled MRSA was cultured with macrophages under different conditions. In macrophages treated with the Day 5 PADM@CZ hydrogel, a significant improvement was observed in viability and an increase in the number of bacteria captured and phagocytosed by bacteria (green fluorescence) was observed compared to the control, PADM, and PADM@Zn groups (**Figure** **5**a, left panel; Figure S7a, Supporting Information, upper panel). In contrast, no differences were observed in cell viability, or the number of bacteria captured or phagocytosed by macrophages following treatment with Day 11 extracts (PADM@CZ, PADM, control, and PADM@Zn) was observed (Figure 5a, right panel; Figure S7b, Supporting Information, upper panel). Meanwhile, flow cytometry results revealed that in contrast to macrophages in the control group, those in the Day 5 PADM@CZ group exhibited an increase in the rate of MRSA phagocytosis from 4.40% to 47.8% (Figure 5b). However, no difference in the rate of MRSA phagocytosis was observed in macrophages treated with the day 11 extracts. Next, the MRSA was cultured with macrophages for 1 h, lysed, and the number of bacteria phagocytosed by macrophages was determined. The trend in the number of bacteria phagocytosed by macrophages in all groups was consistent with confocal laser scanning microscopy (CLSM) observations. That is, a significantly higher number of bacteria was phagocytosed by macrophages in the Day 5 PADM group than in the other groups. Macrophages engulf and kill bacteria through various mechanisms, such as lysosomal degradation.^[^ ^37^ ^]^ Therefore, SPM was used to determine the bactericidal ability of macrophages in the cell‐bacterial co‐culture system. A significantly reduced number of colonies were formed by macrophages in the Day 5 PADM group, indicating the enhanced ability of macrophages in the 5 d PADM group to eliminate bacteria (Figure S7a, Supporting Information, lower panel). Based on the quantitative analysis, a significant decrease in the survival rates of bacteria from 89.5% ± 2.8% (control group) to 38.7% ± 3.6% was observed in the Day 5 PADM group (Figure 5c). Hence, the PADM@CZ hydrogel modulated macrophage activity, affecting its antibacterial ability.

The increase in antimicrobial activity was primarily due to M1 macrophage polarization induced by Cu^2+^ released from PADM@CZ. Mounting evidence has shown that Cu^2+^ is significantly involved in innate immune responses. Furthermore, adequate exogenous supplementation with Cu^2+^ significantly enhances the phagocytic activity and bactericidal ability of immune cells, such as macrophages (Figure 5e).^[^ ^8^, ^38^ ^]^ Hence, low Cu^2+^ concentrations alter the activity and function of the macrophages, thereby decreasing their antibacterial activity of macrophages. This could be the primary cause of the low phagocytic and bactericidal abilities of the macrophages treated with Day 11 PADM hydrogels. However, previous studies have shown that excess inflammatory responses induced by high Cu^2+^ concentrations can compromise the antimicrobial functions of macrophages.^[^ ^39^ ^]^ Therefore, the PADM@CZ hydrogel represents an “immunosuppressive self‐rescuing” biomaterial that initially secretes high levels of Cu^2+^, followed by Zn^2+^ secretions, thus activating macrophages without compromising their functions. However, additional studies are required to determine the precise “immunosuppressive self‐rescuing” mechanism of the PADM@CZ hydrogel. This would aid in determining why PADM@CZ, but not PADM@Zn, can effectively inhibit infection via macrophages.

### 2.5 In Vitro Biocompatibility

Biocompatibility is an important criterion for the clinical use of hydrogels for wound dressing.^[^ ^40^ ^]^ Accordingly, we determined the in vitro biocompatibility of the hydrogels using a Cell Counting Kit (CCK‐8) assay to analyze the morphology of MC3T3‐E1 cells and live/dead staining. CCK‐8 assay results revealed that the hydrogels exerted no cytotoxic effects. Furthermore, the cells in all groups showed similar proliferative behaviors after 3 and 5 d, suggesting that the hydrogels promoted cell proliferation (Figure S8a,b, Supporting Information). Additionally, the morphology of the cells was good (Figure S8c, Supporting Information). After the cells were cultured on different hydrogels for 24 h, live/dead staining was performed. The living cells were stained green, and the nuclei of dead cells were stained red. Figure S8d (Supporting Information) shows that most cells were viable after culturing with the control, PADM, PADM@Zn, or PADM@CZ hydrogels for 24 h. Taken together, these results indicate that the PADM@CZ hydrogels are biocompatible and facilitated cell adhesion, spreading, and proliferation.

### 2.6 Promotion of Bacteria‐Associated Wound Healing by the PADM@CZ Hydrogel

**Figure 6:**
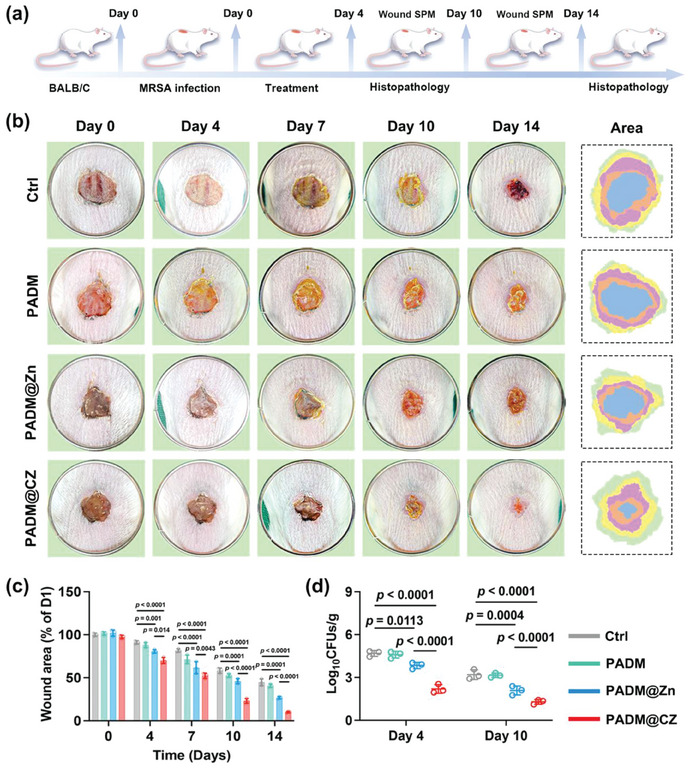
PADM@CZ exhibits enhanced efficacy in promoting the healing of infected wounds in vivo. a) Illustration of the model for mouse excisional wound infection and associated experimental methods. b) Illustrative photographs of the wounds encompassing diverse treatment methodologies, alongside superimposed pictures on Days 0 (blue), 4 (orange), 7 (purple), 10 (yellow), and 14 (green). c) Area of wounds that remained unhealed at different intervals for each group. d) Quantity of bacteria present in the wounds across different treatment groups on Days 4 and 10. c, d) Data presented are expressed as mean ± SD (*n =* 3 per group), with “*n*” denoting biologically independent experiments.

Extended periods of non‐healing wounds increase the likelihood of bacterial infections. Furthermore, such infections may compromise immune function, which adversely affects wound healing, culminating in pervasive infections.^[^ ^41^ ^]^ Hence, expeditious wound healing is imperative for the prevention and treatment of infections. The ADM@CZ hydrogel regulated the phenotype of immune cells in vitro via unique ion‐releasing kinetics, thereby effectively eliminating bacterial infections. Next, we used a mouse model of excisional skin infection to determine the effect of the PADM@CZ hydrogel on wound healing (**Figure** **6**a). The infected wounds gradually healed over time in various treatment groups (Figure 6b). However, significant differences in the extent of infection and the healing rate were observed. Macroscopic infection, foci of tissue necrosis, and pus exudation were observed in the wounds of the control, PADM, and PADM@Zn groups, with severe infection observed on Day 4. However, when the infection was under control, the wounds crusted and healed. In the PADM@CZ group, the wounds were consistently treated, remained clean, and were dry throughout the observation period. No signs of infection or pus formation were observed. Moreover, in mice in the PADM@CZ group, enhanced wound healing was observed, with only 7.1 ± 7.5% unhealed wound detected on Day 14, followed by the PADM@Zn (7.50% ± 0.59%), PADM (7.50% ± 0.59%), and control (14.10% ± 2.33%) groups (Figure 6c). In addition, SPM was performed on Days 4 and 10 to evaluate the bactericidal activity of PADM@CZ against bacteria on the wound surface. A significant decrease in the survival rates of the bacteria was observed upon treatment with the PADM@CZ hydrogel, and relatively complete elimination of the bacteria was observed on Day 10 (Figure 6d; Figure S9, Supporting Information).

**Figure 7:**
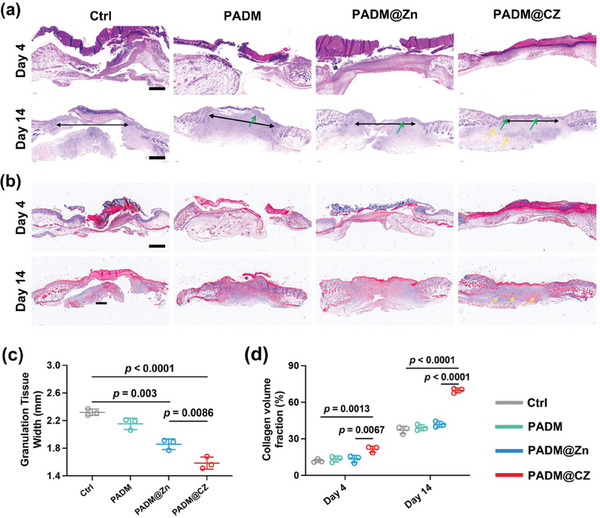
PADM@CZ hydrogel‐induced acceleration of the wound repair. a) H&E staining of wound skin tissues on Days 4 and 14. The green arrows indicate the re‐epithelialization area, and the yellow arrows indicate the newly formed dermal. Scale bar: 500 µm. b) Masson's trichrome staining of wound skin tissues on Days 4 and 14. The yellow arrows indicate the newly formed dermal. Scale bar: 500 µm. c) Corresponding quantitative analysis of the granulation tissue width on Day 14. d) Statistical analysis of collagen deposition during the remolding phase. c,d) Data presented are expressed as mean ± SD (*n =* 3 per group), with “*n*” denoting biologically independent experiments.

Wound healing is a modular process that can be classified into the following phases: hemostasis, inflammation, proliferation, and remodeling.^[^ ^41b^ ^]^ Histological analysis was performed using HE staining; the results were consistent with the wound area (**Figure** **7**a). Additionally, the length of granulation tissue in mice belonging to different groups was measured (Figure 7c). Faster wound contraction was observed in the PADM@CZ group than in the other groups and was prominent during the early stages of wound healing. The statistical analysis of granulation tissue width revealed that wound contraction exhibited a higher rate in the PADM@CZ group compared to the other groups. During the remodeling stage, it is crucial to ensure adequate collagen deposition and remodeling to enhance the tensile strength of the tissue and facilitate optimal healing. Hence, Masson's trichrome staining was performed to examine the emerging collagen fibers. On Day 14, persistent scabs consisting of dried blood, serum, and exudates were evident within the control group, suggesting delayed wound healing (Figure 7b). In contrast, the wounds that received the PADM@CZ hydrogel treatment exhibited a distinct epidermal layer and a notably elevated collagen volume fraction (Figure 7d). This suggests that the regenerating tissue resembled healthy skin and demonstrated enhanced healing efficacy. Angiogenesis is required for nutrients and oxygen supply. Hence, timely and adequate angiogenesis is required for wound healing.^[^ ^42^ ^]^ Angiogenesis analyzed the abundance of neo‐angiogenesis markers, i.e., cell adhesion molecule1 (CD31) and α‐smooth muscle actin (α‐SMA), in wound sections of mouse skin.^[^ ^43^ ^]^ An increase in CD31 and α‐SMA expression was observed in the wound tissues of mice in the PADM@CZ group compared with the other groups (Figure S10, Supporting Information). These results demonstrated the superior wound‐healing ability of the PADM@CZ hydrogels.

### 2.7 Effects of PADM@CZ Hydrogel on Immune Regulation In Vivo

**Figure 8:**
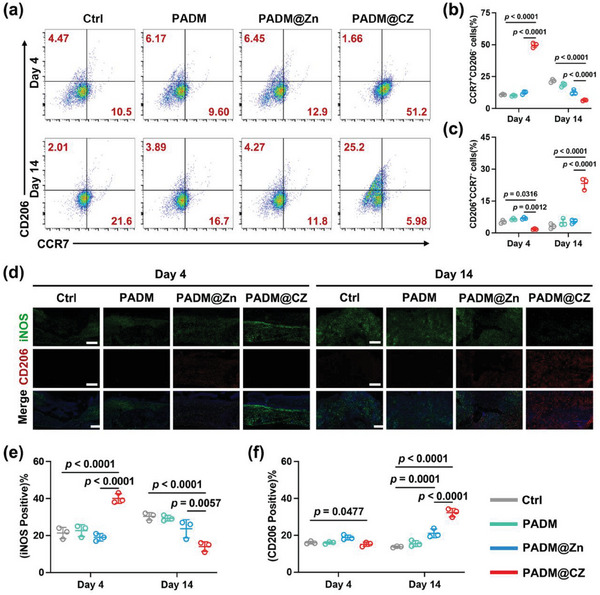
Impact of the PADM@CZ hydrogel on macrophage polarization in vivo. a) Flow cytometry data of M1 and M2 cells retrieved from the wound tissue 4‐ and 14 d post‐injury. b,c) Statistical analysis concerning the proportion of b) M1 and c) M2 macrophages. d) Immunofluorescence staining of tissue sections at the wound site for iNOS (M1 marker) and CD206 (M2 marker) on Days 4 and 14 subsequent to the injury. Scale bar 250 µm. e,f) Statistical analysis concerning the proportion of e) iNOS and f) CD206 positive macrophages. b,c,e,f) Data presented are expressed as mean ± SD (*n =* 3 per group), with “*n*” denoting biologically independent experiments.

Macrophages serve as first responders during tissue damage and are key players in mediating the local immune response to infection. Therefore, we investigated the immunomodulatory effects of the PADM@CZ hydrogel on macrophages at various stages of wound healing. The wounded skin was harvested after 4 and 14 d of treatment under different conditions, and the proportion of macrophages was analyzed using flow cytometry (**Figure** **8**a–c). On Day 4, a significant increase in the proportion of M1 macrophages (CCR7^+^CD206^−^) and a decrease in the proportion of M2 macrophages (CD206^+^CCR7^−^) were observed in the PADM@CZ group. However, by Day 14 of treatment, the PADM@CZ hydrogel induced M2 macrophage polarization. Immunofluorescence was performed on wounded skin at the same time points for iNOS and CD206. The results validated the observed macrophage polarization trend (Figure 8d–f). That is, the PADM@CZ group had the highest M1/M2 ratio on Day 4 and M2/M1 ratio on Day 14 among all groups. Moreover, the observed patterns in inflammation‐related markers, including IL‐6 and TNF‐α (Figure S11, Supporting Information), exhibited congruence with the polarization state of macrophages.

Furthermore, the immune cell composition in the spleen was investigated during wound treatment under various conditions. On Day 4, the CD8^+^/CD4^+^ ratio was significantly higher in the spleens of mice in the PADM@CZ group, indicating that the PADM@CZ hydrogel improved general immunity (Figure S12a,b, Supporting Information). An activated immunity is beneficial to antibacterial effects, which is mainly attributed to Cu^2+^ released by the PADM@CZ hydrogels.^[^ ^44^ ^]^ However, on Day 14, the CD8^+^/CD4^+^ ratio significantly decreased in the PADM@CZ group compared to Day 4, indicating that a sustained release of Zn^2+^ inhibited excess immune activation, thereby accelerating wound healing. In addition, during the pre‐existing period of the PADM@CZ‐treated group (Day 4), a notable augmentation in Th1 (CD4^+^CCR5^+^) infiltration and a substantial reduction in Tregs (CD4^+^Foxp3^+^) infiltration were observed (Figure S12c–f, Supporting Information). These findings underscore the significance of PADM@CZ in modulating the equilibrium between immune cells that promote activation and those that suppress immune response, thereby augmenting the effectiveness of humoral immunity. Furthermore, HE staining revealed no abnormalities or damage to mouse major organs, indicating that the PADM@CZ exhibited good biocompatibility in vivo (Figure S13, Supporting Information). Taken together, these results suggest that the PADM@CZ hydrogel possesses good antibacterial and immunomodulatory abilities in vivo and accelerates wound healing by promoting angiogenesis.

The challenge of effectively treating infection‐associated wounds has been a persistent concern for researchers. Despite the development of numerous therapeutic approaches, the presence of stubborn bacteria and dysregulated immune responses significantly impede their sustained effectiveness.^[^ ^45^ ^]^ Macrophages, which play a pivotal role in the processing of internal antigens and the initiation of adaptive immunity against bacterial pathogens.^[^ ^46^ ^]^ M1 macrophages are crucial in the initiation of pro‐inflammatory immune responses, bacterial phagocytosis, antimicrobial agent release, and bacterial antigen presentation.^[^ ^47^ ^]^ Nevertheless, the persistence of infection prompts the polarization of immune cells toward an anti‐inflammatory phenotype. This transition from M1 to M2 macrophages is marked by impaired antigen processing and presentation.^[^ ^45a^ ^]^ The bioactive ions released by PADM@CZ primarily consist of Cu^2+^ and Zn^2+^, both of which hold significant significance in immune activity. The biological impacts of Cu^2+^ are intricately linked to its concentration; at suitable levels, Cu^2+^ demonstrates efficacy in eradicating bacteria and stimulating angiogenesis, thereby expediting the process of wound healing. Nevertheless, an excessive immune response can lead to a disruption of redox equilibrium, exacerbating immune disorders and consequently perpetuating the persistence of infection. Research has demonstrated that an imbalanced redox microenvironment at the infection site not only facilitates the formation of biofilms but also hinders the antimicrobial immune response.^[^ ^48^ ^]^ Conversely, the interaction between Zn^2+^ and immune cells promotes the development of an anti‐inflammatory microenvironment, alongside the beneficial angiogenic and tissue repair attributes of Zn^2+^. Hence, in the current investigation, it was observed that PADM@CZ exhibited a sequential release of Cu^2+^ and Zn^2+^. By conducting RNA sequencing on RAW264.7 macrophages, it was discovered that PADM@CZ effectively stimulated antibacterial pathways, particularly the NOD‐like receptor signaling pathway, during the initial phase (Day 5). The in vitro assays demonstrated that PADM@CZ could induce a pro‐inflammatory phenotype and enhance the phagocytosis of RAW264.7 cells. The in vivo flow cytometry analysis demonstrated that PADM@CZ exhibited the ability to induce macrophage M1 phenotype polarization and enhance the CD8T/CD4T ratio during the early stage (Day 4), which plays a crucial role in bacterial presentation and immune activation. However, it is noteworthy that during the late stage, PADM@CZ primarily modulates the immune system employing Zn^2+^, aiming to prevent excessive immune activation and mitigate the risk of exacerbating immune disorders. Overall, this research will inspire continued innovation in the design of biomaterials with immunostimulatory effects until this new approach to immunotherapy is implemented.

## 3 Conclusion

Herein, we developed a PADM@CZ hydrogel to promote wound healing following infection by releasing Cu^2+^ and Zn^2+^ sequentially. In vitro, the PADM@CZ hydrogel exhibited a good dual‐temporal bidirectional immunomodulatory effect and regulates M1/M2 macrophage polarization from Days 5 to 11 by alternately activating the NOD‐like pathway and inhibiting MAPK signaling pathways. Furthermore, the superior immunomodulatory, antibacterial, and transient direct bactericidal abilities of the PADM@CZ hydrogel exerted synergistic anti‐infection effects in the wound infection model. However, our study did not comprehensively examine the mobilization and functions of immune cells, such as DC cells and NK cells, in the initial phase of wound healing in infected wounds, in addition to macrophages. Moreover, the molecular mechanisms underlying these processes were not explored. In our upcoming research phase, we will specifically focus on investigating these aspects to gain a better understanding of the factors that contribute to the enhancement of healing in infected wounds through the use of biomaterials. Nevertheless, this novel dual‐temporal bidirectional immunomodulatory hydrogel exhibits good antibacterial and wound healing‐ effects and can be applied to design and synthesize biomaterials that regulate immune responses based on the immune profile of a target disease.

## 4 Experimental Section

### Synthesis and Characterization of Cu–Zn BGns

A modified Stöber method was used to synthesize the Cu–Zn BGns.^[^ ^49^ ^]^ First, TEOS was mixed in ethanol (solution A) with ammonia, deionized water (DI‐H_2_O), and ethanol (solution B) with stirring. Calcium nitrate tetrahydrate was added after 30 min. Subsequently, zinc nitrate was added, and the reaction was allowed to proceed for 90 min before centrifugation at 8000 rpm for 15 min. The deposits were collected, washed with DI‐H_2_O thrice, and calcined at 700 °C for 2 h to obtain Zn‐BGns. Next, the Zn‐BGns were dissolved in DI‐H_2_O (solution C) with ammonia, CTAB, ethanol, and DI‐H_2_O (solution D) by stirring for 30 min, followed by a reaction with TEOS for 15 min. Next, we added Cu/ascorbic acid complex precursors,^[^ ^50^ ^]^ and the mixture was stirred for 24 h. The colloids were centrifuged at 8000 rpm for 15 min, collected, washed with DI‐H_2_O twice and ethanol once, and dried at 60 °C overnight. Finally, Cu–Zn BGns were calcined at 700 °C for 2 h. The morphology and constituents of nanoparticles were determined using SEM (S4800, Hitachi, Japan) and TEM (JEM2100, Hitachi, Japan) with EDS. The chemical composition and state of the nanoparticles were analyzed using XRD (Bruker, Billerica, USA), XPS (ESCALAB 250Xi, Thermo Fisher, USA), and FTIR (Magna‐IR 750, Thermo Fisher, USA). DLS measurements were performed by a Wyatt Mobius DLS instrument.

### Synthesis of PADM@CZ Hydrogel

After rinsing with sterile water for 3 h, the porcine skin tissue was subjected to repeated freeze/thaw cycle procedures with liquid nitrogen. The tissues were then agitated at 120 rpm and 25 °C to remove the subcutaneous tissue. The samples were incubated with 0.1% Triton X‐100/PBS (v/v) for 12 h and 0.1% sodium dodecyl sulfate for 6 h. Subsequently, they were lyophilized, powdered, and digested with pepsin in an acidic environment (pH 2‐3) for 10 min.^[^ ^51^ ^]^ Cu–Zn BGns solution was added dropwise, and the mixture was quickly stirred. The gel was stored at 4 °C and digested for 2 h until it became translucent and viscous. Next, PBS was added, the osmotic pressure was adjusted, and the pH was adjusted to 7‐8 using precooled 10 m NaOH. Finally, to prepare PADM@CZ hydrogels, the gels were incubated at 37 °C for 20 min, freeze‐dried, and compressed. The final products obtained, namely PADM@CZ (1%), PADM@CZ (5%), and PADM@CZ (10%), were contingent upon the mass differentials between the added particles and the lyophilized hydrogel powder. The PADM and PADM@Zn hydrogels were prepared using the same method, however, Cu–Zn BGns were not added or replaced with Zn‐BGns.

### Characterization of PADM@CZ Hydrogels: Characterization

HE and DAPI staining were performed to assess the removal of cellular and nuclear particles. The samples were dehydrated using a critical‐point dryer, and the structural characteristics of the hydrogels were analyzed using SEM. Finally, the chemical compositions of the materials were characterized using XPS, XRD, and FTIR spectroscopy.

### Mechanical Property Tests

To assess the mechanical properties of the hydrogels, their compression capabilities were analyzed using a universal testing machine (Instron 5567, USA). Cylindrical hydrogels measuring 10 mm in height and 5 mm in diameter were fabricated for testing. Compression tests were performed at a controlled speed of 5 mm min^−1^ until a maximum strain of 75% was achieved. Rheological tests were performed using a rheometer (MARS 60, USA) to measure the mechanical properties of the hydrogels. Hydrogel circles with diameters of ≈1.5 cm were prepared for testing. The test was performed with gap values of 1–2 mm, 1 Hz frequency, 1% strain, and a 130 s time oscillation scan.

### Water Retention

PADM, PADM@Zn, and PADM@CZ hydrogels were incubated at 37 °C. Their masses were measured at set time points until no change in mass was observed. Water retention in the hydrogels was calculated using Equation (1):

(1) $$\mathrm{Water}\;\mathrm{retention}\;\mathrm{rate}=W_{2}/W_{1}\times 100\%$$

where *W*_2_ corresponds to the weight of the hydrogel at each time point, and *W*_1_ corresponds to its initial weight.

### Biodegradability of Hydrogels

Next, the biodegradability of the PADM, PADM@Zn, and PADM@CZ hydrogels were determined under various conditions. The samples were incubated in microcentrifuge tubes at different temperatures (25 °C, 37 °C, or 42 °C) and stirred slowly, along with subsequent regular observation. The liquid from the samples was aspirated, and the remaining samples were weighed. Next, hydrogels were solidified and incubated in PBS, followed by incubation with or without trypsin at 37 °C. The solution was removed from the centrifuge tubes and the hydrogels were rinsed with PBS daily. Finally, the samples were dried and weighed.

### Measurement of Ion Release

To investigate the ion release behavior of the PADM@CZ hydrogel, the same mass of each hydrogel was placed in 10 mL of simulated body fluid (SBF, PH1820, Scientific Phygene, China). Next, the release medium was completely removed and equal volumes of SBF were changed daily. The Cu and Zn ion concentrations were measured using an inductively coupled plasma atomic emission spectrometer (X Series 2, Thermo Fisher Scientific, USA). The PADM and PADM@Zn hydrogels, which were used as control groups, were evaluated using the same method.

### Cells and Bacteria

Mouse‐derived macrophages (RAW264.7) and MC3T3‐E1 osteoblast precursor cells were obtained from the Shanghai Institute of Cell Biology. Cells were grown in Dulbecco's modified Eagle's medium (DMEM) supplemented with 10% fetal bovine serum (FBS) and 1% penicillin/streptomycin. MRSA (ATCC 43300) was purchased from the American Type Culture Collection and was cultured in tryptic soy broth (TSB; Hopebio).

### Preparation of Sample Extracts

The PADM, PADM@Zn, and PADM@CZ hydrogels were sliced into small sections measuring 10 × 10 × 5 mm and immersed in DMEM supplemented with 10% FBS and 1% penicillin/streptomycin. The cultures were maintained at 37 °C for 14 d with daily medium renewal. To explore the distinct impacts of each sample on cells, extracts were collected daily during the incubation period and stored at 4 °C for subsequent analysis.

### In Vitro Antibacterial Tests: Direct Antibacterial Assay

MRSA frozen cultures were incubated in TSB at 37 °C overnight on a shaker, diluted at a 1:10,000 ratio, and cultured until the logarithmic growth phase. Subsequently, 500 µL of bacterial suspensions containing 1 × 10^6^ colony forming units (CFUs) mL^−1^ were cultured in hydrogel extracts (Days 1, 2, 3, 4, and 5). The effects of the materials on bacterial growth were evaluated using a growth curve assay and a microplate reader. Absorbance was measured at 600 nm from 0 to 24 h. All experiments were conducted in triplicates and repeated three times. Next, we co‐cultivated bacteria with a diverse set of hydrogels for 24 h, followed by SPM and quantification of the CFUs on agar plates. Additionally, we analyzed the antibacterial activity by live/dead staining. Briefly, we added 500 µL of combined dye (Syto9 and PI) in different samples and cultured them for 15 min. Bacteria were collected on glass slides and visualized under a fluorescence microscope. Apoptosis and damage to cell membranes were analyzed; detached bacteria were stained for 15 min, centrifuged, washed with cold PBS, and collected. The bacteria were analyzed by flow cytometry.

### Estimation of ROS Levels

DCFH‐DA (a ROS probe) was used to measure ROS levels in cells. The bacteria were cultured for 24 h and incubated with 10 × 10^−6^ m DCFH‐DA for 30 min. A fluorescence microscope was used to measure ROS levels in the cells. The fluorescence intensity was measured with the aid of a microplate reader at *λ*_ex_/*λ*_em_ = 485 nm/525 nm. The ROS levels in bacteria from all groups were measured in triplicates. Additionally, the bacteria were stained and analyzed using a flow cytometer.

### Membrane Damage and Leakage Assay

Bacteria were cocultured for 24 h, stained with 2.5 µg mL^−1^ PI for 30 min, centrifuged, washed, and analyzed by flow cytometry. Similarly, bacteria were incubated with PI for 30 min, and the fluorescence intensity of these samples was measured at *λ*_ex_/*λ*_em_ = 535 nm/615 nm using a microplate reader. Next, we evaluated the leakage of cellular contents under various treatments. To this end, the bacterial supernatant was centrifuged at a rate of 8000 × *g* for 10 min, and the absorbance was measured at 260 nm (A260, nucleic acids) and 280 nm (A280, proteins) using a spectrophotometer. All experiments were performed in triplicate.

### TEM

TEM was performed to analyze changes in the ultrastructure of the bacterial membranes. Briefly, bacteria (1 × 10^6^ CFUs mL^−1^) were treated with various hydrogels for 24 h, resuspended in 1 mL of fixative, and treated with osmium tetroxide. The bacteria were then dehydrated using an ethanol gradient, embedded and cut using a diamond knife. The resulting sections were placed on mesh copper mesh grids, stained with lead citrate, and visualized by TEM.

### In Vitro Immunomodulatory Assays: Enzyme‐Linked Immunosorbent Assay (ELISA)

RAW 264.7 cells were cultured with hydrogel extracts (Days 5 and 11) for 24 h and the cell culture supernatants were harvested for measuring IL‐1β, TNF‐α, IL‐6, and IL‐10 levels using ELISA (MultiSciences Biotech, China).

### Gene Expression

Microarray analysis was performed to determine the gene expression patterns of macrophages. Briefly, RAW 264.7 cells were seeded at a density of 5 × 10^5^ cells per well and cultured with PADM@CZ hydrogel extracts prepared on Days 5 and 11 for 24 h. Next, total cellular RNA was isolated using TRIzol reagent based on the manufacturer's guidelines. RNA‐seq and bioinformatics analysis were performed by Shanghai Novelbio Ltd. The KEGG pathway and Gene Ontology enrichment analyses were performed on the DEGs.

### RT‐PCR

RAW 264.7 cells were cultured and collected after 24 h. Total RNA was isolated using TRIzol. Gene expressions were normalized to housekeeping genes (*ACTB*). The primer sequences are shown in Table S1 (Supporting Information).

### Immunofluorescence

Briefly, cells were incubated with Day 5 or 11 PADM, PADM@Zn, or PADM@CZ hydrogel extracts for one day. Subsequently, the cells were collected, fixed, and blocked before staining with antibodies against iNOS (ab210823) and CD206 (ab64693). Next, cells were incubated with corresponding secondary antibodies and counterstained with DAPI for 1 h. The cells were observed and photographed using CLSM.

### Flow Cytometry Assay

Flow cytometry was performed using antibodies against CCR7 and CD206 to determine the proportion of M1 and M2 macrophages. Briefly, macrophages were cultured with Days 5 or 11 hydrogel extracts for 24 h, blocked for 30 min, and stained with allophycocyanin (APC)‐conjugated CD206 and phycoerythrin (PE)/Cy7‐conjugated CCR7. Finally, the cells were washed, resuspended in 500 µL of PBS, and analyzed by flow cytometry.

### Macrophage‐Mediated Bactericidal Assay

Fluorescence‐labeled MRSA was used to quantitatively analyze the phagocytosis of bacteria by macrophages treated with various hydrogels. Briefly, 1 mL of 2 × 10^9^ CFU mL^−1^ MRSA was stained using 400 µL of 1 µg mL^−1^ CFDA‐SE for 30 min in the dark. Excess dye was removed, and the fluorescence‐labeled MRSA was resuspended in PBS for subsequent experiments. Next, 1 × 10^5^ RAW264.7 cells per well were treated with Days 5 or 11 hydrogel extracts from the four groups for 24 h, harvested, and cultured in new well plates. Next, RAW264.7 cells were cultured with ≈1 × 10^7^ CFU mL^−1^ fluorescence‐labeled MRSA for 2 h and stained with Dil (Beyotime) for 15 min, following the manufacturer's instructions. Finally, the cells were washed and imaged using CLSM. Following the same procedure, the efficiency of macrophage bacterial phagocytosis was evaluated by flow cytometry without staining the cell membrane. Additionally, to determine the bactericidal effect of macrophages on MRSA, RAW264.7 cells were lysed using 1% Triton X‐100 to release the bacteria, which were counted via the SPM. Thereafter, to determine the phagocytosis of bacteria by RAW264.7 cells, fluorescent‐bacteria and RAW264.7 cells were cocultured for 2 h, and the extracellular fluorescent‐MRSA was quenched using trypan blue. Phagocytosis of MRSA by macrophages was measured at excitation/emission wavelengths of 485/520 nm with a microplate reader.

### In Vitro Cytocompatibility

: Following the manufacturer's instructions, cytotoxicity was analyzed by staining the cells with a live/dead staining kit. Briefly, MC3T3‐E1 cells were seeded on the hydrogels and cultured for 24 h. Additionally, a CCK‐8 assay was performed to evaluate the biocompatibility of the hydrogels. Briefly, cells were seeded in 96‐well plates at a density of 5 × 10^3^ cells per well and cultured for 24 h, and 100 µL of the different hydrogel extracts were added to the wells. At pre‐set time points, the medium was replaced with a CCK‐8 mixture solution (10% CCK‐8 solution and 90% DMEM medium) and incubated for 2 h. Finally, the absorbance was measured at 450 nm using a microplate reader (BioTek). The measurements were repeated thrice for each group. For live‐cell staining, the cells were washed and incubated with calcein and PI (for dead cells) for 30 min.

### Cell Morphology

MC3T3‐E1cells were cultured for 1 d, washed three times with PBS, fixed with paraformaldehyde, and permeabilized using Triton X‐100. Next, actin was stained with fluorescein isothiocyanate‐phalloidin. Finally, the nuclei were stained with DAPI and observed via CLSM.

### In Vivo Evaluation: Mouse Wound Model and Treatment

The Animal Ethics and Welfare Committee of Changzheng Hospital approved all animal experiments (approval number 2023‐564). All surgical procedures were conducted following the established guidelines. Male ICR mice (4–5 weeks old, 18–20 g) were used to create a wound infection mouse model. First, the dorsal surfaces of the mice were depilated. Next, 8 mm diameter wounds were created, and 10 µL of 2  ×  10^9^ CFU mL^−1^ bacterial solution was inoculated. The next day, the mice were randomly categorized into the control (Ctrl), PADM, PADM@Zn, or PADM@CZ (*n* = 10 per group) groups. Briefly, MRSA‐infected wounds were treated with control (PBS), PADM, PADM@Zn, or PADM@CZ (this treatment was performed only once in the entire experimental cycle). The mice were provided with food and water ad libitum. Moisture‐, temperature‐, and light‐controlled (12 h light‐dark cycle) microinsulators were used to house the mice. Digital photographs were captured daily, and the area of the wounds was measured using the “Image J” software. The percentage of the wound area was calculated using Equation (2):

(2) $$\begin{array}{ccc} \mathrm{Scratch}\;\mathrm{open}\;\mathrm{area}\;\left(\%\right) & = & (\mathrm{remaining}\;\mathrm{wound}\;\mathrm{area}/\\ & & \mathrm{original}\;\mathrm{wound}\;\mathrm{area}\;\mathrm{vehicle})\times 100 \end{array}$$

### Microbiological and Histological Analyses

Mice from each group were euthanized on days 5 and 11 post‐surgery, and infected tissues were collected and homogenized with 5 mL of PBS in order to evaluate the bacterial load. Next, the wounds with surrounding tissues were harvested for histological evaluation, including HE staining and Masson's trichrome staining. Immunofluorescence was performed to determine the expression of endogenous iNOS, CD206, CD31, and α‐SMA. Immunohistochemistry was used performed to measure IL‐6 and TNF‐α levels.

To evaluate immune infiltrate phenotyping, spleen cells were treated with CD45 (AF488; Biolegend, cat. no. 160305, clone S18009D), CD3 (PE; Biolegend, cat. no. 100205, clone 17A2), CD4 (BV605; Biolegend, cat. no. 100547, clone RM4‐5), CD8a (PercP/Cy5.5; Biolegend, cat. no. 100733, clone 53‐6.7), CCR5 (PercP/Cy5.5; Biolegend, cat. no. 107016, clone HM‐CCR5), CD8a (BV421; Biolegend, cat. no. 126419, clone MF‐14) antibodies on ice. After 30 min, the cells were washed twice with PBS and subjected to flow cytometry. Finally, major organs such as the heart, spleen, lung, liver, and kidney were isolated for further examinations.

### Statistical Analysis

Numeric data were reported as mean ± standard deviation (SD), unless specified otherwise. A two‐tailed Student's *t*‐test was used to compare two groups, while multiple comparisons were conducted using one‐way analysis of variance (ANOVA) with Tukey's post hoc test. Excel 2016 and GraphPad Prism 9 were used for all calculations and statistical analyses. Statistical significance was set at *P < 0.05*.

## Conflict of Interest

The authors declare no conflict of interest.

## Author Contributions

S.H., S.L., and Q.L. contributed equally to this work. S.H.: conceptualization, methodology, investigation, writing‐original draft, data curation. S.L.: conceptualization, methodology, validation, investigation. Q.L.: conceptualization, methodology, investigation, data curation. E.X.: investigation, data curation, methodology. L.M.: methodology, investigation. X.M.: methodology, investigation. Z.X.: conceptualization, resources. C.Z.: conceptualization, methodology, investigation, funding acquisition. X.L.: conceptualization, resources, supervision, funding acquisition, writing‐review & editing. G.X.: conceptualization, resources, supervision, project administration, funding acquisition, writing‐review & editing.

## Supporting information

Supporting Information

## Data Availability

The data that support the findings of this study are available from the corresponding author upon reasonable request.
