# Supplementary material for: Copper–Zinc‐Doped Bilayer Bioactive Glasses Loaded Hydrogel with Spatiotemporal Immunomodulation Supports MRSA‐Infected Wound Healing
Source: Adv Sci (Weinh). 2023 Nov 30;11(5):2302674. doi: 10.1002/advs.202302674 (PMC10837387; doi:10.1002/advs.202302674)
Supplement: Supplementary file 1 — Supporting Information [file ADVS-11-2302674-s001.pdf]

ADVANCED SCIENCE
Open Access

Supporting Information

for *Adv. Sci.*, DOI 10.1002/advs.202302674

Copper–Zinc-Doped Bilayer Bioactive Glasses Loaded Hydrogel with Spatiotemporal Immunomodulation Supports MRSA-Infected Wound Healing

Shicheng Huo, Shu Liu, Qianqian Liu, En Xie, Licai Miao, Xiangyu Meng, Zihao Xu, Chun Zhou, Xuesong Liu* and Guohua Xu**

Supporting Information

Copper-Zinc doped bilayer bioactive glasses loaded hydrogel with spatiotemporal immunomodulation supports MRSA-infected wound healing

*Shicheng Huo[a, 1], Shu Liu[d, 1], Qianqian Liu[e, 1], En Xie[f], Licai Miao[g], Xiangyu Meng[g], Zihao Xu[g], Chun Zhou[c, *], Xuesong Liu[b, *], Guohua Xu[a, *]*

[a] Department of Orthopedic Surgery, Spine Center, Changzheng Hospital, Navy Medical University, Shanghai 200003, China

[b] Department of Ultrasound, Renji Hospital, School of Medicine, Shanghai Jiaotong

[c] Orthpaedic Trauma, Department of Orthopedics, Renji Hospital, School of Medicine, Shanghai Jiao Tong University

[d] Department of Spine Surgery, Changhai Hospital, Navy Military Medical University, 168 Changhai Road, Shanghai 200433, China

[e] Department of Medical Record Statistics, Sichuan Provincia People's Hospital, University of Electronic Science and Technology of China, Chengdu, China.

[f] Key Laboratory for Ultrafine Materials of Ministry of Education, East China University of Science and Technology, Shanghai 200237, China

[g] Department of Orthopedics Trauma, Shanghai Changhai Hospital, Naval Medical University, Shanghai 200433, China.

*Corresponding authors:

E-mail: xuguohuamail@smmu.edu.cn (Guohua Xu), cedarsky@163.com (Xuesong Liu), zoucnu@163.com (Chun Zhou)

Shicheng Huo, Shu Liu, and Qianqian Liu contributed equally to this work.

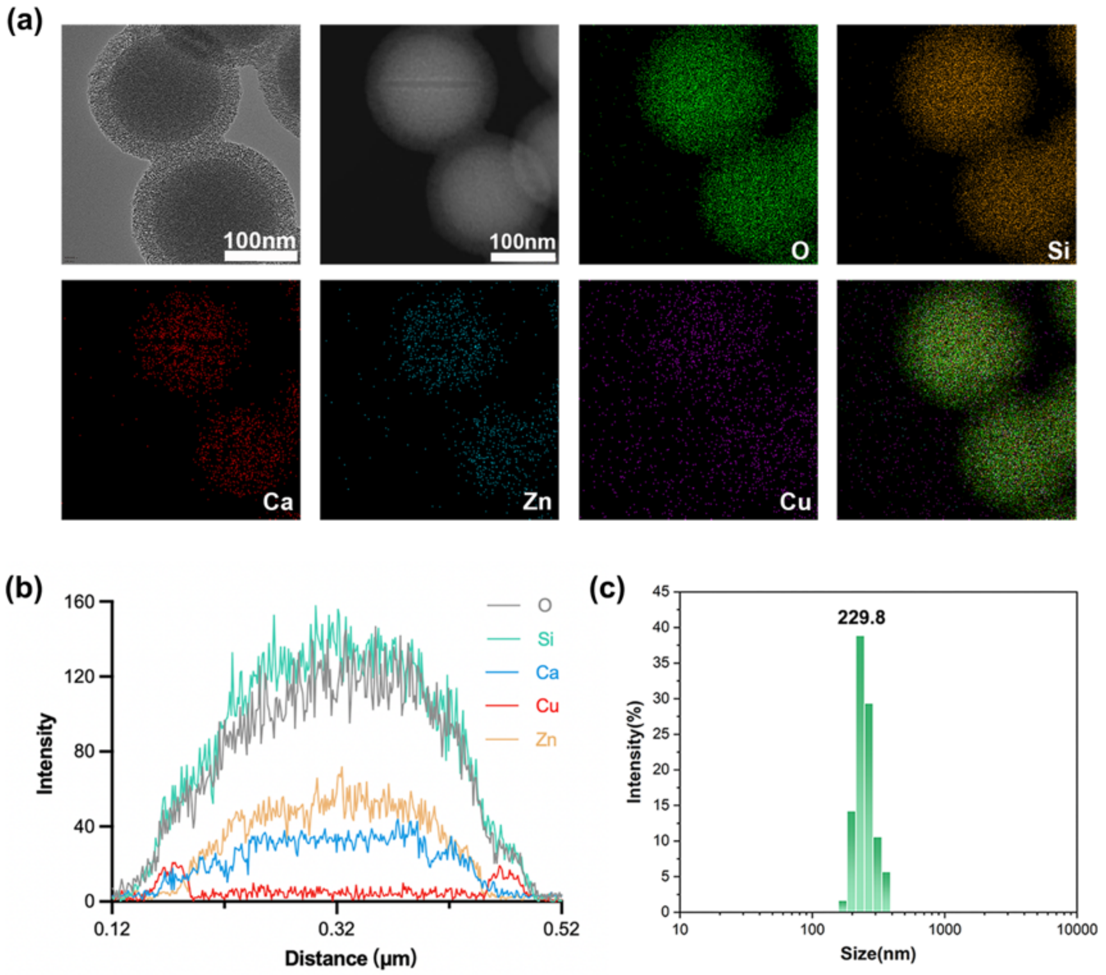

Figure S1. Characterization of Cu-Zn BGns. (**a**) TEM and EDS images of Cu-Zn BGns. Scale bar: 200 nm. (**b**) EDS line scan of Cu-Zn BGns. (**c**) DLS results of Cu-Zn BGns.

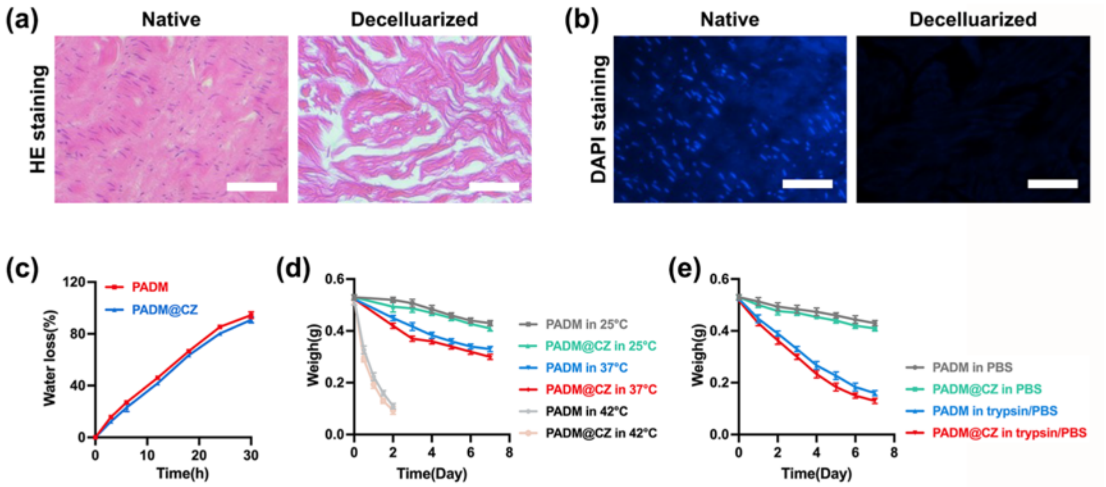

Figure S2. Preparation and Characterization of PADM hydrogels. (**a-b**) HE (**a**) and DAPI (**b**) stainings of the porcine skin extracellular matrix showing that cells are completely cleared. Scale bar: 500 μm (HE staining); 400 μm (DAPI staining). (**c**) PADM and PADM@CZ

hydrogels exhibited negligibly different water retentions. (**d**) PADM and PADM@CZ hydrogels degraded at 25, 37, and 42 °C. (**e**) PADM and PADM@CZ hydrogels degraded with or without trypsin.

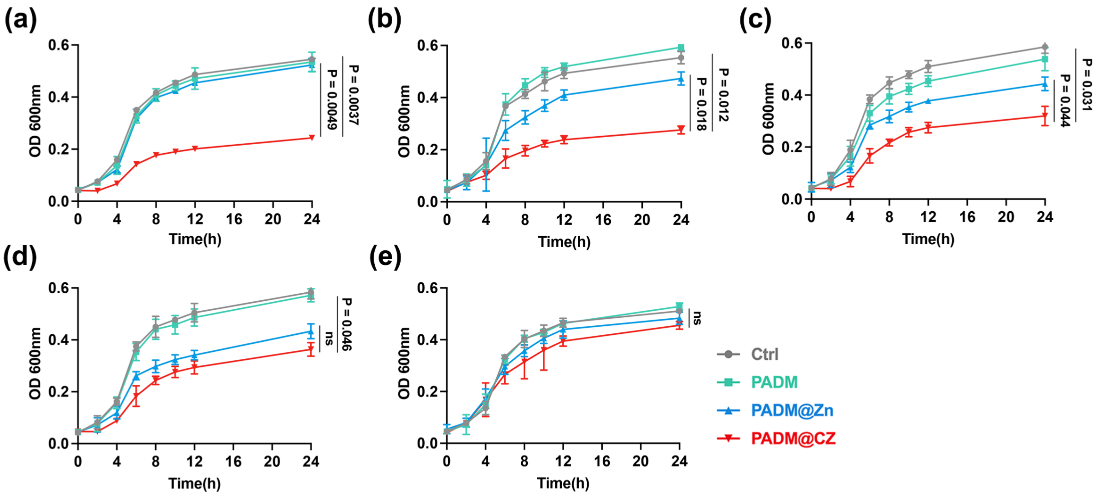

Figure S3. The time-kill kinetics assay of various samples against MRSA. (**a-e**) The growth suppression effects of various samples of 1-day (**a**), 2-day (**b**), 3-day (**c**), 4-day (**d**), and 5-day (**e**) on bacteria.

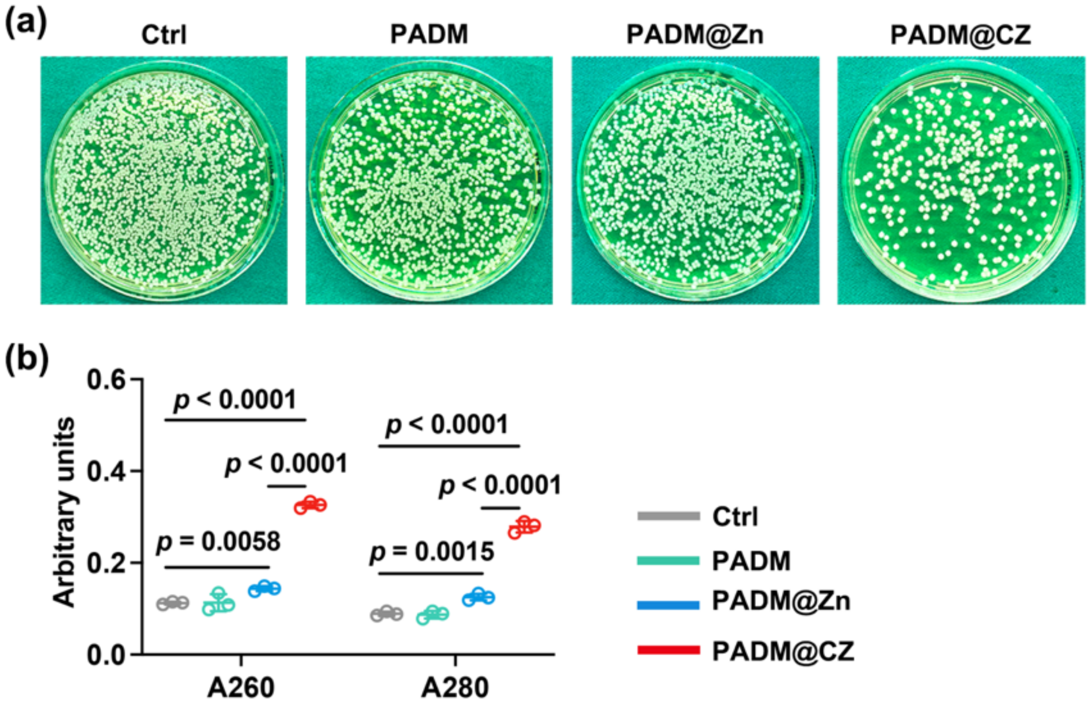

Figure S4. (**a**) Representative images of the bacterial colonies formed by the bacteria cultured

with various samples after 24 h. (**b**) Leakage of cell contents after exposure to various PADM based hydrogels was investigated by monitoring the absorbance of extracellular material at 260 nm (A260) and 280 nm (A280) corresponding to nucleic acids and proteins, respectively. (**b**) Data presented are expressed as mean ± SD (*n* = *3*/group), with 'n' denoting biologically independent experiments.

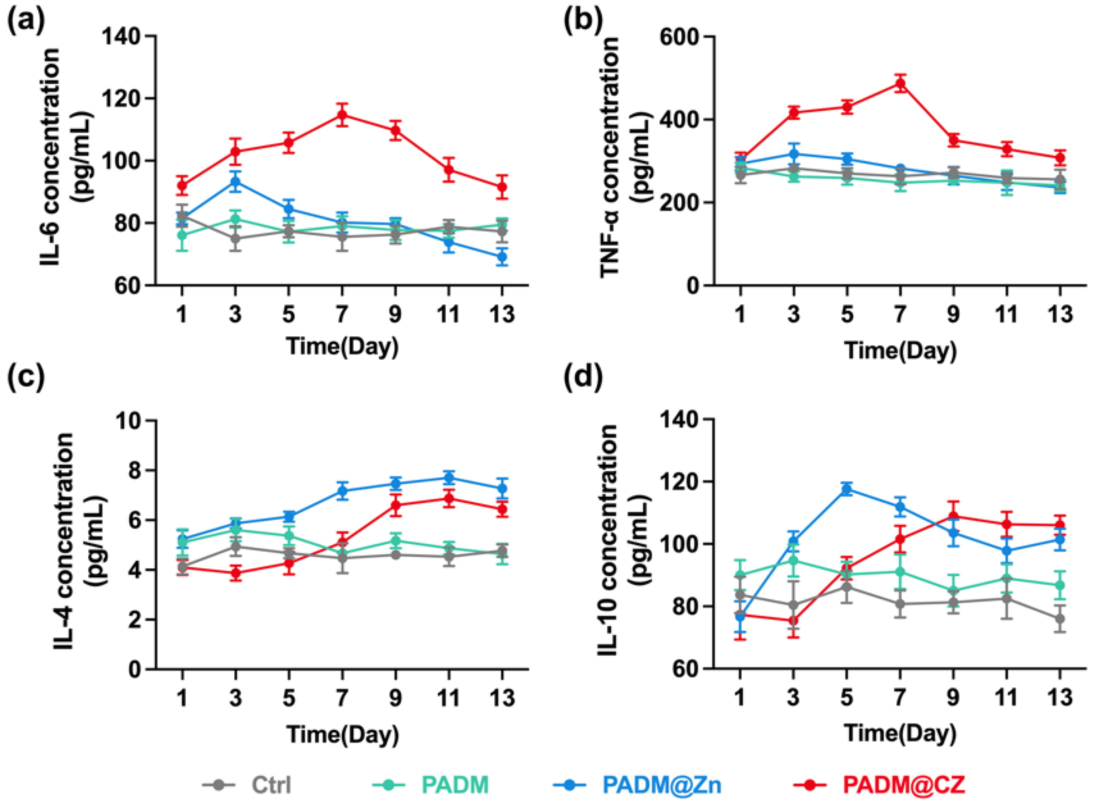

Figure S5. Inflammatory cytokines secretion of RAW264.7 cells cultured in the extracts of various samples of 1, 3, 5, 7, 9, 11, 13 day for 24 h. (**a, b**) The concentrations of pro-inflammatory cytokines IL-6 (**a**) and TNF-α (**b**). (**c, d**) The concentrations of anti-inflammatory cytokines IL-4 (**c**) and IL-10 (**d**).

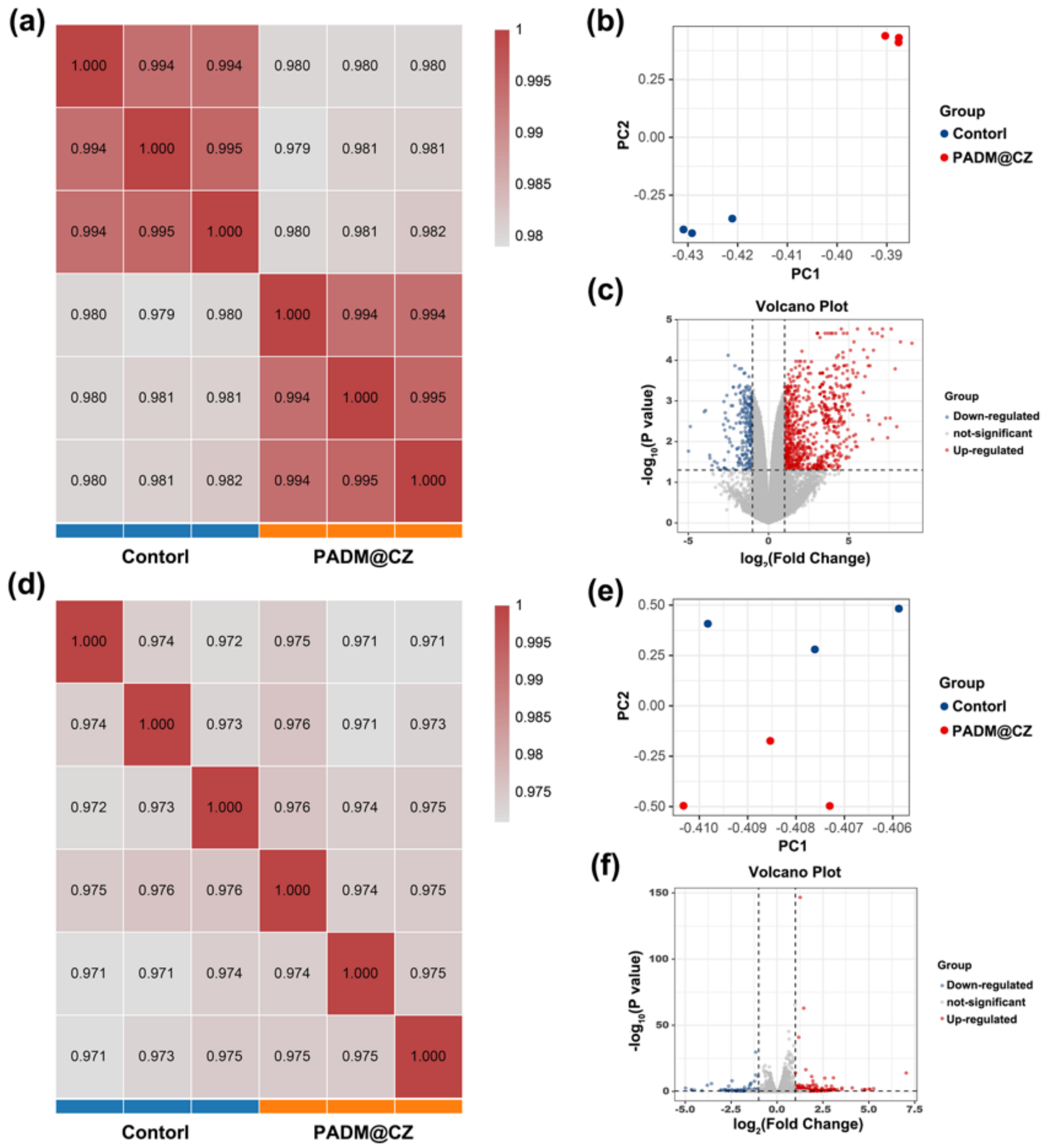

Figure S6. Results of transcriptome sequencing. (**a, d**) Correlation analysis of gene expression levels between samples in cells co-cultured with PACM@CZ samples on days 5 (**a**) and days 11 (**d**) for 24 h. (**b, e**) PCA of the global genes in cells co-cultured with PACM@CZ samples on days 5 (**b**) and days 11 (**e**) for 24 h. (**c, f**) Volcano plot displaying the differentially expressed genes (fold change ≥ 2 and $p < 0.05$) in cells co-cultured with PACM@CZ samples on days 5 (**c**) and days 11 (**f**) for 24 h.

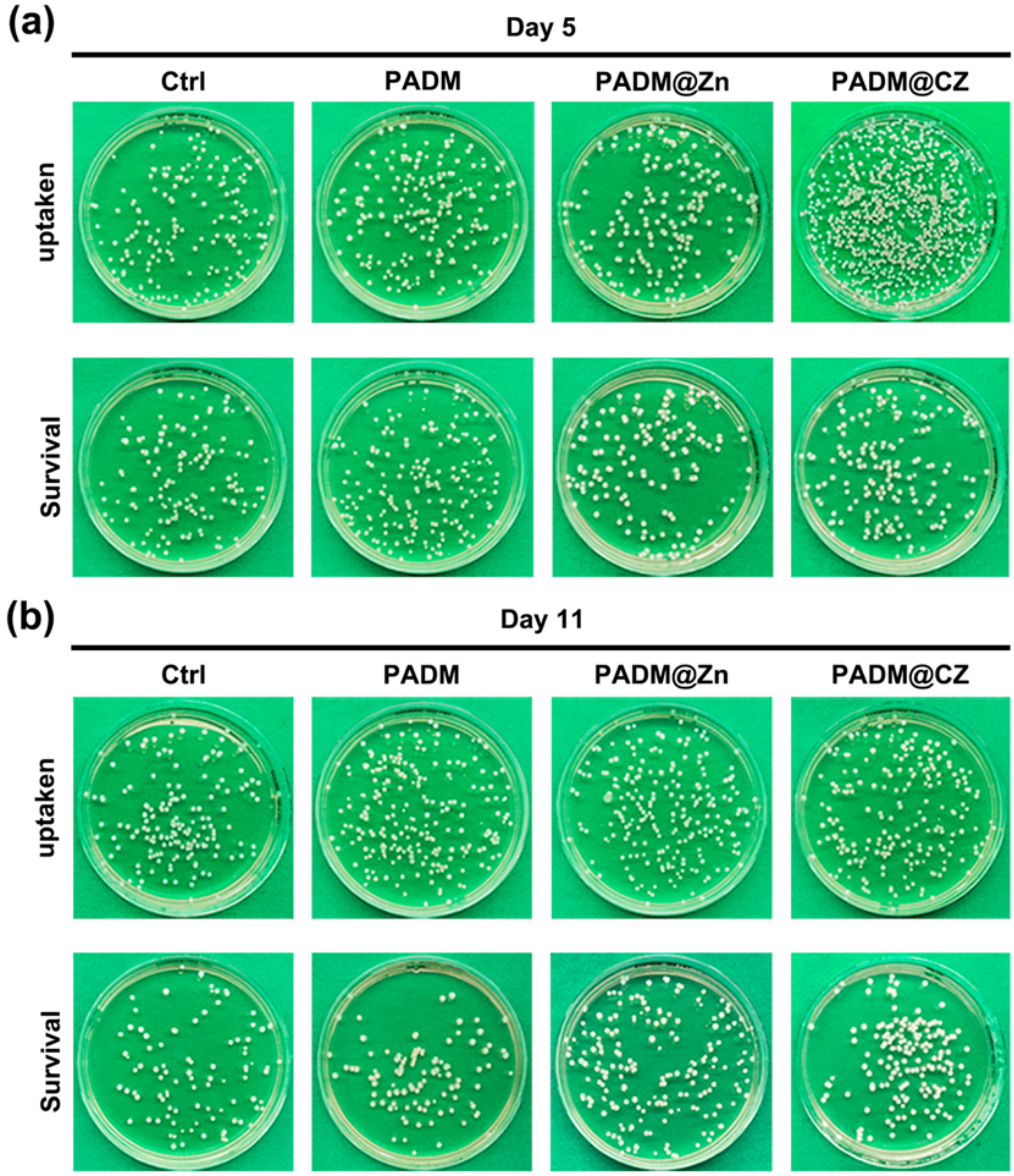

Figure S7. *In vitro* indirect antimicrobial SPM results for Days 5 and 11 samples. (**a**) Representative images of the bacterial colonies formed by the MRSA phagocytosed by macrophages cultured with Day 5 samples (upper panel); Representative images of the bacterial colonies formed by the MRSA survived the macrophage phagocytosis cultured with Day 5 samples (lower panel). (**b**) Representative images of the bacterial colonies formed by the MRSA phagocytosed by macrophages cultured with Day 11 samples (upper panel); Representative images of the bacterial colonies formed by the MRSA survived the macrophage phagocytosis cultured with Day 11 samples (lower panel).

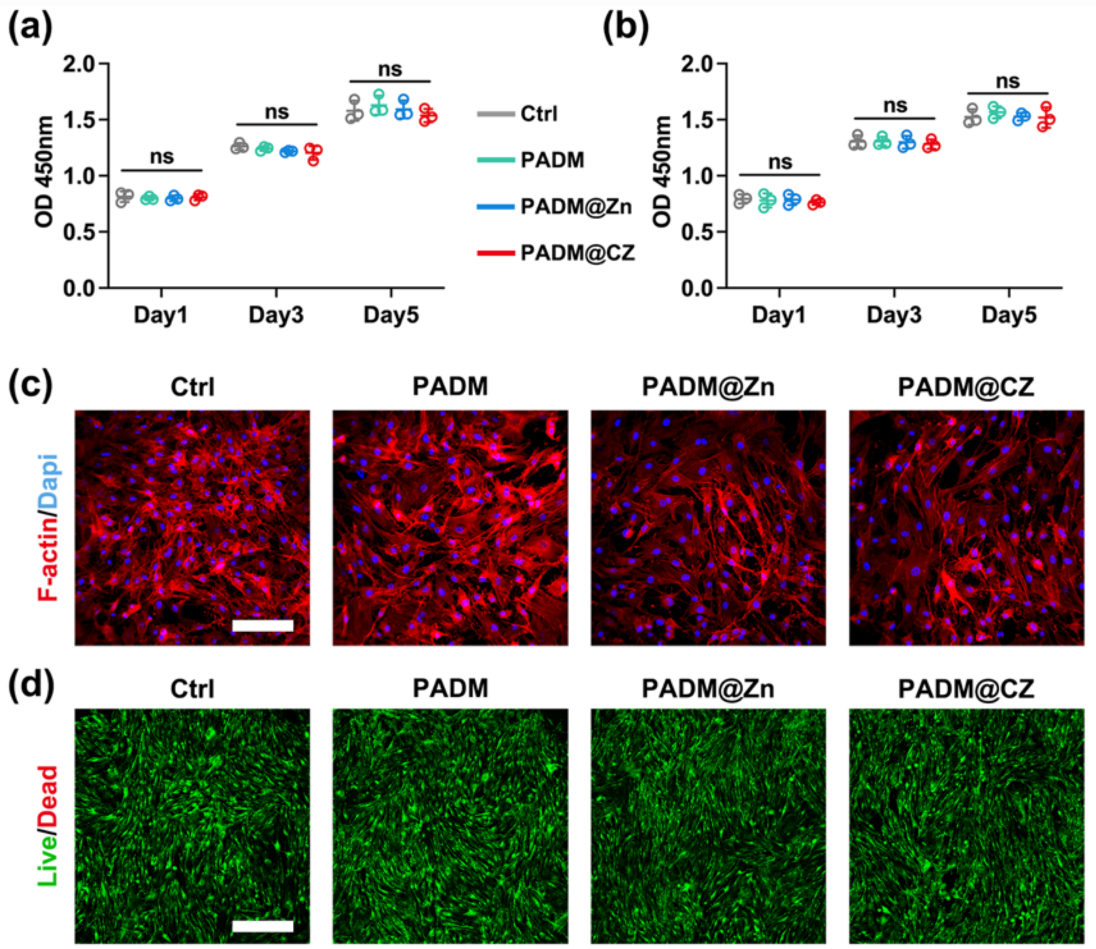

Figure S8. *In vitro* cytocompatibility of various samples. (**a-b**) Cytotoxic of RAW 264.6 (**a**) MC3T3-E1 (**b**) treated with different samples for 1, 3, or 5 days, as tested by CCK-8 assays. (**c**) CLSM analysis of the morphology of MC3T3-E co-cultured on hydrogels for 24 h. Scale bar: 100 μm. (**d**) Confocal microscopy images of a live/dead assay of MC3T3-E1 cells co-cultured on hydrogels for 24 h. Scale bar: 200 μm. (**a**), (**b**) Data presented are expressed as mean ± SD (*n* = *3*/group), with 'n' denoting biologically independent experiments.

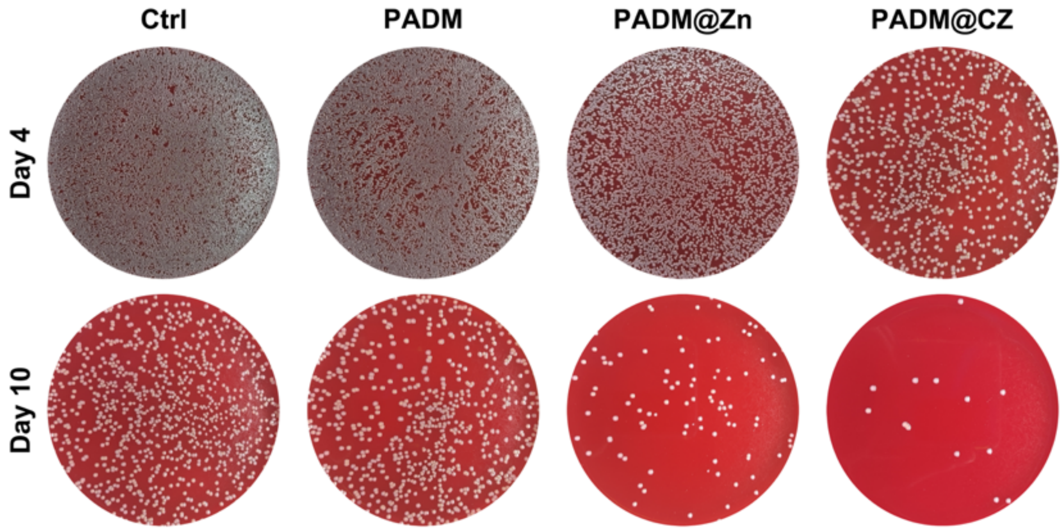

Figure S9. The bacterial burden of wound skin tissues after 4 (upper panel) and 10 days (lower panel) after surgery.

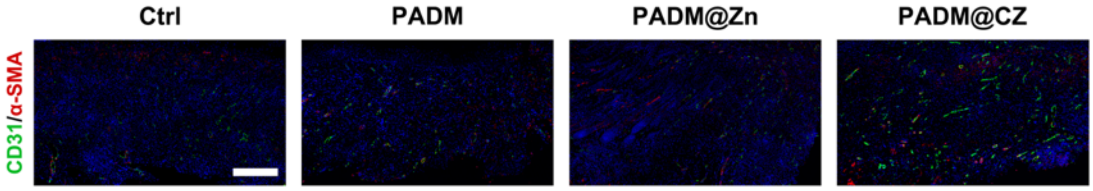

Figure S10. Representative immunofluorescence staining images of CD31 (Green) and α-SMA (Red) of wound skin tissues on day 14. Scale bar: 100 μm.

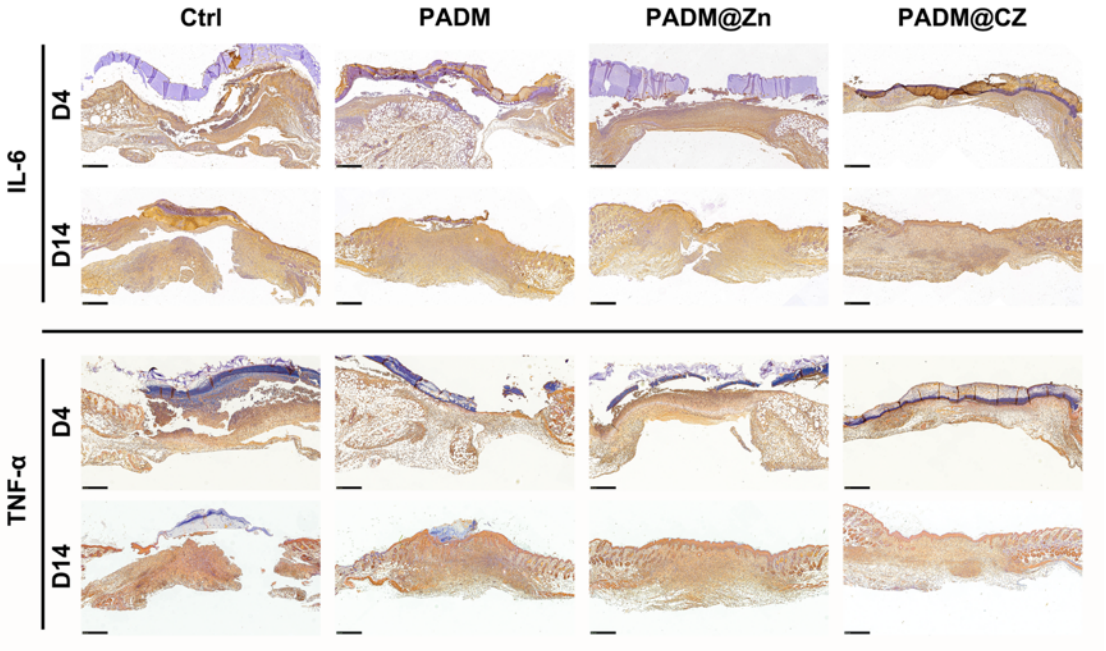

Figure S11. The immunohistochemical staining of IL-6 and TNF-α in the skin tissues of the wounded area after 4- and 14- days. Scale bar 200 μm.

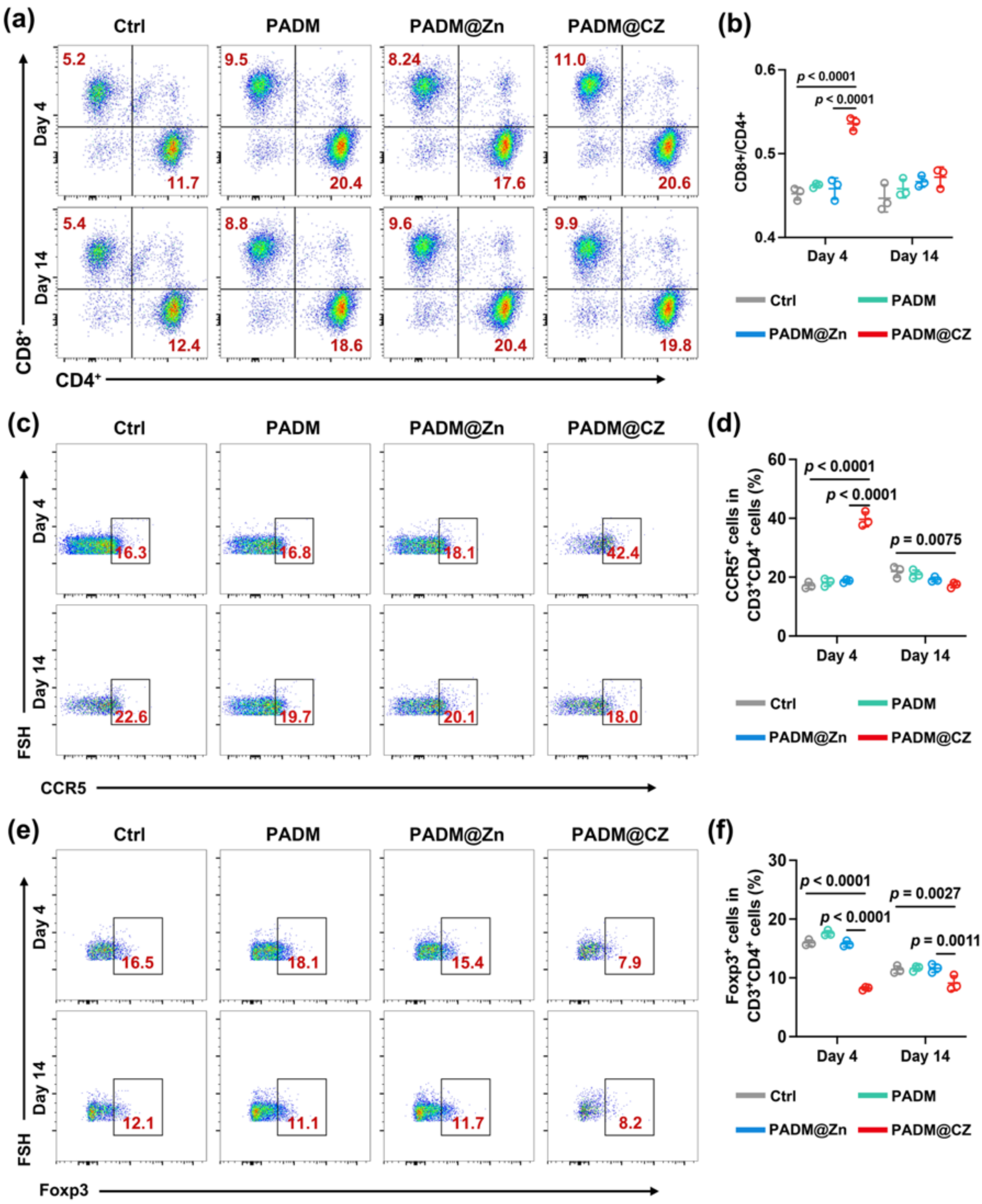

Figure S12. Impact of the PADM@CZ hydrogel on immune regulation *in vivo*. (**a, b**) Flow cytometry analysis of $CD4^+$ and $CD8^+$ T cells (**a**), and $CD8^+/CD4^+$ ratios (**b**) in the spleens of mice subjected to diverse treatments over varying time intervals. (**c, d**) Representative flow cytometric analysis and quantification of Th1 ($CD4^+CCR5^+$) proportion in the spleens of mice.

(**e, f**) Representative flow cytometric analysis and quantification of Treg ($CD4^{+}Foxp3^{+}$) proportion in the spleens of mice. (**b**), (**d**), (**f**) Data presented are expressed as mean ± SD (*n* = *3*/group), with 'n' denoting biologically independent experiments.

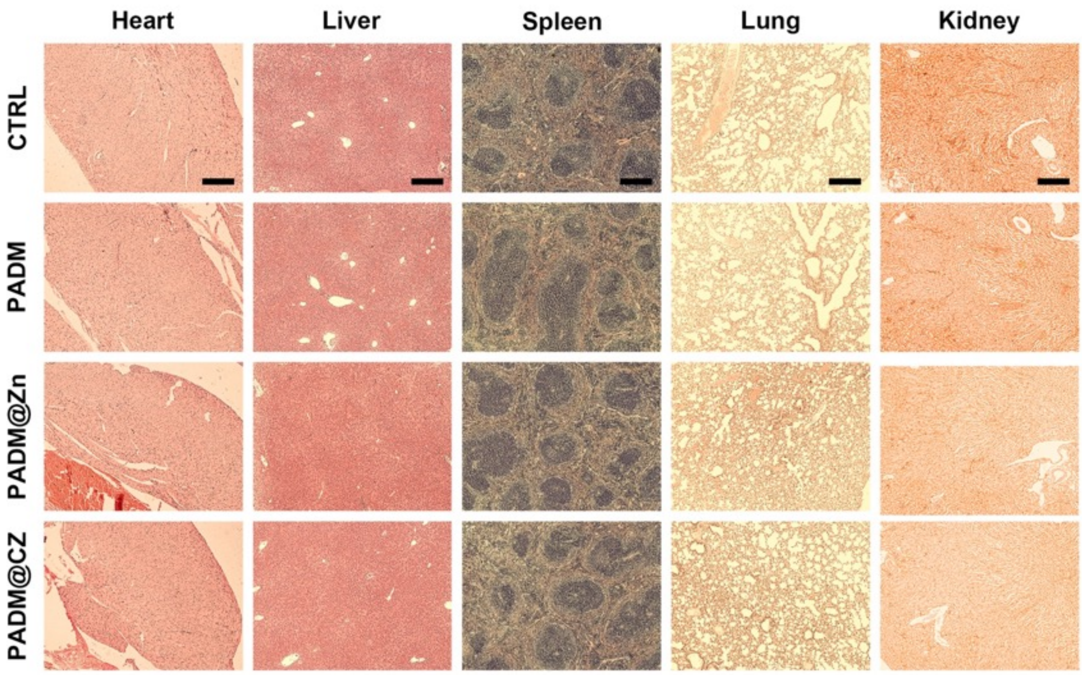

Figure S13. Histological evaluation of major organs (heart, liver, spleen, and kidney). Scale bar 100 μm**.**

Table S1 Primers used in this study.

Gene and primer direction	Primer sequence (5′to 3′)
Mouse IL-6	
Forward	TAGTCCTTCCTACCCCAATTTCC
Reverse	TTGGTCCTTAGCCACTCCTTC
Mouse IL-10	
Forward	GGCTGGACGAGAGCCGAACG
Reverse	CCCGGGGTGTAGGCACCACT
Mouse CD206	
Forward	TACTTGGACGGATAGATGGAGG
Reverse	CATAGAAAGGAATCCACGCAGT
Mouse CCR7	
Forward	GGTGGCTCTCCTTGTCATTTTC

Reverse	AGGTTGAGCAGGTAGGTATCCG
Mouse Actin	
Forward	GGCTGTATTCCCCTCCATCG
Reverse	CCAGTTGGTAACAATGCCATGT
